# Surfactant-Free RAFT Emulsion Polymerization of Styrene Using Thermoresponsive macroRAFT Agents: Towards Smart Well-Defined Block Copolymers with High Molecular Weights

**DOI:** 10.3390/polym9120668

**Published:** 2017-12-03

**Authors:** Steffen Eggers, Volker Abetz

**Affiliations:** 1 Department of Physical Chemistry, University of Hamburg, Grindelallee 117, 20146 Hamburg, Germany; steffen.eggers@chemie.uni-hamburg.de; 2 Institute of Polymer Research, Helmholtz-Zentrum Geesthacht, Max-Planck-Straße 1, 21502 Geesthacht, Germany

**Keywords:** reversible addition-fragmentation chain transfer polymerization, emulsion polymerization, polymerization-induced self-assembly, block copolymers, micelles, stimuli-responsiveness, thermoresponsiveness

## Abstract

The combination of reversible addition–fragmentation chain transfer (RAFT) and emulsion polymerization has recently attracted much attention as a synthetic tool for high-molecular-weight block copolymers and their micellar nano-objects. Up to recently, though, the use of thermoresponsive polymers as both macroRAFT agents and latex stabilizers was impossible in aqueous media due to their hydrophobicity at the usually high polymerization temperatures. In this work, we present a straightforward surfactant-free RAFT emulsion polymerization to obtain thermoresponsive styrenic block copolymers with molecular weights of around 100 kDa and their well-defined latexes. The stability of the aqueous latexes is achieved by adding 20 vol % of the cosolvent 1,4-dioxane (DOX), increasing the phase transition temperature (PTT) of the used thermoresponsive poly(*N*-acryloylpyrrolidine) (PAPy) macroRAFT agents above the polymerization temperature. Furthermore, this cosolvent approach is combined with the use of poly(*N,N*-dimethylacrylamide)-*block*-poly(*N*-acryloylpiperidine-*co*-*N*-acryloylpyrrolidine) (PDMA-*b*-P(APi-*co*-APy)) as the macroRAFT agent owning a short stabilizing PDMA end block and a widely adjustable PTT of the P(APi-*co*-APy) block in between 4 and 47 °C. The temperature-induced collapse of the latter under emulsion polymerization conditions leads to the formation of RAFT nanoreactors, which allows for a very fast chain growth of the polystyrene (PS) block. In dynamic light scattering (DLS), as well as cryo-transmission electron microscopy (cryoTEM), moreover, all created latexes indeed reveal a high (temperature) stability and a reversible collapse of the thermoresponsive coronal block upon heating. Hence, this paper pioneers a versatile way towards amphiphilic thermoresponsive high-molecular-weight block copolymers and their nano-objects with tailored corona switchability.

## 1. Introduction

While reversible addition–fragmentation chain transfer (RAFT) polymerizations have nowadays entered various fields of chemistry [1], industry [2] and medicine [3,4], it still appears challenging to synthesize well-controlled high-molecular-weight polymers from slowly-propagating monomers; so-called low-*k*_p_ monomers [5]. One highly relevant class of such polymers is the styrenics, the chain growth of which is slow due to the resonance stabilization of their growing radicals [6,7]. However, aqueous RAFT emulsion polymerization has recently proven to be a versatile and green tool to tackle this challenge [8,9,10]. By using hydrophilic latex-stabilizing poly(acrylic acid) (PAA) [8,9] or poly(*N*-hydroxyethyl acrylamide-*co*-poly(ethylene glycol) methyl ether acrylate) (P(HEAA-*co*-PEGA)) [10] macroRAFT agents, it has been demonstrated that synthesizing styrenic block copolymers with molecular weights above 100 or even 1000 kDa is possible, indeed.

Besides its potential for synthesizing high-molecular-weight block copolymers and allowing for high polymer loadings in the latexes up to 50 wt % [11,12,13], RAFT emulsion polymerization (both growing block and monomer are insoluble in the polymerization medium) and its relative RAFT dispersion polymerization (the monomer in contrast to the growing solvophobic polymer block is well-soluble) have attracted much attention in the past eight years or so because of a related process named ‘polymerization-induced self-assembly’ (PISA). Achievements in this field have recently been reviewed for instance by Armes et al. [14], Truong et al. [15] and Boyer et al. [16]. PISA simultaneously takes place with the chain extension of the solvophilic macroRAFT agent/macro-stabilizer with a solvophobic block as the latter becomes insoluble at a certain block length and therefore induces micellization [17,18,19,20]. While the chain growth is slow before the micellar nucleation and (in the case of emulsion polymerizations) dependent on the diffusion of solvophobic monomer molecules from the monomer droplets into the solvent phase (Stage 1), the polymerization rate massively increases after micellization (Stage 2). This is caused by the compartmentalization (meaning spatial isolation) of the growing radicals inside the micelle cores reducing bimolar termination reactions compared to conventional RAFT solution polymerizations and thus allowing for high chain lengths. Constant monomer diffusion from the monomer droplets into the micelle cores due to the rapid monomer consumption therein and the confined space moreover lead to a high local monomer concentration, which further accelerates the chain growth. In dispersion polymerizations, the polymerization rate in Stage 2 is actually increased by a factor of roughly five [21], while the rate acceleration is much more pronounced for styrene polymerizing in aqueous emulsion [10]. However, PISA in emulsion polymerizations of styrene almost exclusively ends up in the formation of spherical micelles even if thermodynamics favor other morphologies, i.e., cylindrical structures (worms, rods) or vesicles, depending on the volume fractions of the different blocks and thus packing parameter [14,22,23]. The full range of thermodynamically expected morphologies and even more kinetically-trapped structures can, however, be obtained via PISA in dispersion polymerizations, given a high polymer concentration and a short solvophilic stabilizing block enabling micelle fusion [21,24,25,26,27].

One inherent problem of aqueous RAFT emulsion polymerizations emerges when the hydrophilic stabilizing block is supposed to be thermoresponsive, as those blocks often become insoluble above a certain temperature, the so-called cloud point or phase transition temperature (PTT). This PTT is usually well below the polymerization temperature of 65–80 °C, and thermoresponsive polymers hence appear to be unsuitable macroRAFT agents/macro-stabilizers. They are, nevertheless, a highly interesting class of materials, for instance as chemical valves [28,29] or drug delivery systems [30,31,32]. Therefore, the groups of Davis [33,34] and Monteiro [35,36,37] tried to overcome the lack of stabilizing ability of thermoresponsive macroRAFT agents in aqueous emulsion polymerizations by adding the low-molecular-weight anionic surfactant sodium dodecyl sulfate (SDS). Upon cooling of the created latex after completion of the polymerization to temperatures below the PTT of the thermoresponsive block, its rapidly changing hydrophilicity leads to a temperature-induced morphological transformation (TIMT) if an appropriate plasticizer for the core-forming block is added. This TIMT transfers originally spherical nano-objects into various structures, such as worms (‘filomicelles’), vesicles and others. However, the use of a surfactant like SDS has certain disadvantages. For example, it has to be removed from the polymerization mixtures by either centrifugation or dialysis for some applications (at best above the PTT of the thermoresponsive block) [37,38]; the latexes are less stable, e.g., at higher solids above ca. 10 wt % [37,39]; and SDS makes the deoxygenation of the polymerization mixtures by N_2_-sparging quite nasty due to pronounced foam formation. To the best of our knowledge, the only examples dealing with surfactant-free aqueous emulsion polymerizations using thermoresponsive macroRAFT agents/macro-stabilizers have been published by Monteiro et al. [40,41,42] and Davis’s group [43]. The former utilized a nanoreactor approach, making use of an irreversibly terminated poly(*N*,*N*-dimethylacrylamide)-*block*-poly(*N*-isopropylacrylamide) (PDMA-*b*-PNIPAm) diblock copolymer in combination with a PNIPAm macroRAFT agent [40,42] and of a PDMA-*b*-PNIPAm macroRAFT agent [41], respectively. Truong and Davis et al. presented a poly(di(ethylene glycol) methyl ether methacrylate-*co*-*N*-(2-hydroxypropyl) methacrylamide-*co*-poly(ethylene glycol) methyl ether methacrylate) (P(DEGMA-*co*-HPMA-*co*-PEGMA)) macroRAFT agent as a biocompatible thermoresponsive macro-stabilizer [43]. In that system, 21 ethylene glycol units in the PEGMA side chains were actually necessary to stabilize the thermoresponsively collapsed P(DEGMA-*co*-HPMA) part at the polymerization temperature of 70 °C. While by the nanoreactor approach, also high-molecular-weight styrenic block copolymers >100 kDa were accessible, Davis et al. have reported thermoresponsive styrenic block copolymers with rather low molecular weights <20 kDa. All three publications furthermore do not discuss the thermoresponsiveness of the obtained nano-objects in detail.

**Figure 1:**
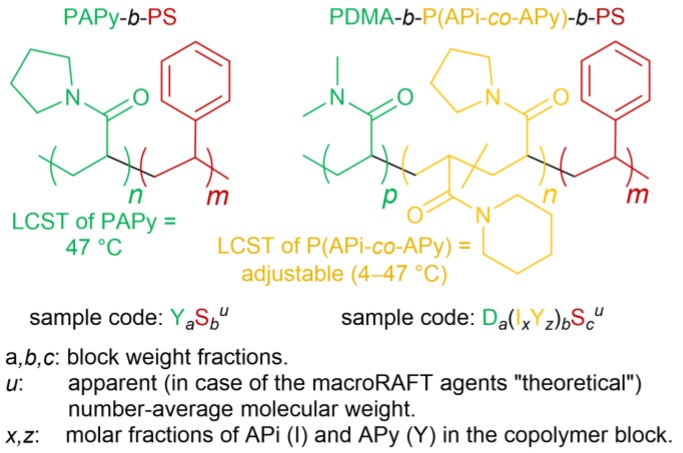
Chemical structures of the block copolymers being investigated in this work. The lower critical solution temperatures (LCSTs) of the thermoresponsive blocks are furthermore given [46]. It is focused on the synthesis via reversible addition–fragmentation chain transfer (RAFT) emulsion polymerization and the related self-assembly. The targeted molecular weights are up to >100 kDa, the targeted poly(*N*-acryloylpyrrolidine) (PAPy)/polystyrene (PS) and poly(*N,N*-dimethylacrylamide) PDMA/poly(*N*-acryloylpiperidine-*co*-*N*-acryloylpyrrolidine) (P(APi-*co*-APy))/PS weight ratios are 20/80 and 2/20/80, respectively. A key for the sample codes used in this work is given at the bottom of the figure.

Taking the latter examples into account, it becomes clear that the straightforward synthesis of high-molecular-weight thermoresponsive block copolymers with major polystyrene (PS) blocks is still highly challenging. Up to now, it usually includes at least one anionic polymerization step for the controlled synthesis of the PS block [44,45]. In this work, we present a fast, feasible and, as much as possible, sustainable pathway to thermoresponsive high-molecular-weight styrenic block copolymers and their nano-objects using surfactant-free water-based RAFT emulsion polymerization. For that, we choose poly(*N*-acryloylpyrrolidine) (PAPy) and PDMA-*b*-P(APi-*co*-APy) with a very short hydrophilic PDMA block (PDMA/P(APi-*co*-APy) ≈ 1/10, *w*/*w*) as thermoresponsive macroRAFT agents and efficient stabilizers (Figure 1). We actually like to play with this comonomer couple of APi and APy because the lower critical solution temperature (LCST) of their random copolymers is linearly adjustable between 4 °C (LCST of pure poly(*N*-acryloylpiperidine) (PAPi)) and 47 °C (LCST of pure PAPy) by simply copolymerizing both monomers in a proper ratio [46,47]. To lift the PTT of the unstabilized macroRAFT agents above the polymerization temperature, water with slight amounts (20 vol %) of DOX as a cosolvent is chosen as the polymerization medium. Our goal is to deliver a procedure being adaptable for the preparation of various amphiphilic high-molecular-weight thermoresponsive block copolymers and their nano-objects via RAFT emulsion polymerization.

## 2. Materials and Methods 

### 2.1. Materials

Acryloyl chloride (>97%, Sigma-Aldrich (Munich, Germany), contained 400 ppm phenothiazine as stabilizer, stored at 4 °C), anhydrous dichloromethane (DCM) (99.9%, extra dry, Acros Organics (Geel, Belgium)), 4,4′-azobis(4-cyanovaleric acid) (ACVA) (>98%, Sigma-Aldrich, stored at 4 °C), 2-(dodecylthiocarbonothioylthio)propionic acid (DTPA) (97%, Sigma-Aldrich, stored at 4 °C) and *N,N*-dimethylformamide (DMF) (>99.5%, VWR Chemicals (Darmstadt, Germany)) were used as received. Pyrrolidine (>99%, Acros Organics) and piperidine (99%, Acros Organics) were stored at 4 °C over molecular sieves (mesh size = 4 Å). Styrene (99%, Grüssing (Filsum, Germany), stored at 4 °C) and *N,N*-dimethylacrylamide (DMA) (99%, Sigma-Aldrich, stored at 4 °C) were freshly percolated through a column of basic alumina (>98%, Brockmann I, Sigma-Aldrich) prior to use to remove the inhibitor methyl ether hydroquinone. DOX (99%, Grüssing) was stored over KOH pellets (>85%, Merck (Darmstadt, Germany)) and freshly percolated through a basic alumina column prior to use to remove peroxides. Ultrapure water (MilliQ quality, resistivity >18.2 MΩ·cm^−1^) was obtained from a Millipore (Darmstadt, Germany) MilliQ water purification system. All other chemicals were used as received in at least analytical grade.

#### 2.1.1. Synthesis of APy and APi

The syntheses of APy and APi were conducted as described elsewhere [46,47,48].

#### 2.1.2. Synthesis of the macroRAFT Agents/Macro-Stabilizers

The syntheses of the macroRAFT agents were conducted in 10-mL screw-capped vials sealed with bored poly(propylene) caps and silicone/poly(tetrafluoroethylene) septums. The heating was performed in a thermoshaker at 300 rpm.

##### Synthesis of the PAPy macroRAFT Agents/Macro-Stabilizers

A typical experiment for the RAFT polymerization of APy was conducted as follows: DTPA (8.8 mg, 25 µmol, 1.0 eq) and APy (995 mg, 7.95 mmol, 317 eq) were dissolved in DOX/H_2_O (6/4, *v*/*v*) (3.0 mL), and DMF (167 µL) was added as an internal standard for determination of the monomer conversion. ACVA (47 µg, 0.17 µmol, 0.007 eq) in DOX/H_2_O (6/4, *v*/*v*) (50 µL) was added, and a reference sample was taken for NMR. The solution was deoxygenated by N_2_-bubbling for 15 min in an ice bath and shaken at 70 °C. After 105 min, the polymerization was quenched by ice cooling and exposure to air. An NMR sample was taken for conversion determination, and the volatiles were removed at 30 °C under reduced pressure. The residue was dissolved in acetone (5 mL) and the polymer precipitated in ice-cold *n*-hexane (100 mL). This procedure was repeated further three times until the polymer was obtained as a yellow powder, which was dried in vacuo at room temperature for 24 h. Monomer conversion = 44%. Size-exclusion chromatography (SEC): ${\bar{M}}_{n,\mathrm{app}}$ = 12 kDa (${\bar{M}}_{n,\mathrm{th}}$ = 18 kDa), *Ð* = 1.29. For further analytical data, see Table 1 and Figure S1b.

##### Synthesis of the Short Chain PDMA macroRAFT Agent

DTPA (116 mg, 331 µmol, 1.0 eq), ACVA (0.9 mg, 3 µmol, 0.01 eq), DMA (982 mg, 9.91 mmol, 30 eq) and DMF (167 µL) as the internal standard were dissolved in DOX (3.0 mL), and an NMR reference sample was taken. The solution was deoxygenated by N_2_-bubbling for 15 min in an ice bath and shaken at 70 °C for 90 min. The polymerization was quenched by ice cooling and exposure to air. An NMR sample was taken for determination of the DMA conversion, the solution diluted by addition of tetrahydrofuran (THF) (7 mL) and the polymer precipitated in ice-cold *n*-hexane (200 mL). The sticky precipitate was redissolved in THF (10 mL) and again precipitated in ice-cold *n*-hexane (200 mL). The PDMA macroRAFT agent was obtained as a yellow powder which was dried in vacuo at room temperature for 24 h. Monomer conversion = 77%. SEC: ${\bar{M}}_{n,\mathrm{app}}$ = 1.7 kDa (${\bar{M}}_{n,\mathrm{th}}$ = 2.6 kDa), *Ð* = 1.24. For further analytical data, see Table 2.

##### Synthesis of the PDMA-*b*-P(APi-*co*-APy) macroRAFT Agents/Macro-Stabilizers

A typical procedure for the RAFT copolymerization of APi and APy using a PDMA macroRAFT agent was as follows: The PDMA macroRAFT agent (45 mg, 17 µmol, 1.0 eq), APi (762 mg, 5.47 mmol, 320 eq), APy (246 mg, 1.97 mmol, 115 eq) and DMF (167 µL) as the internal standard were dissolved in DOX/H_2_O (6/4, *v*/*v*) (3.0 mL). ACVA (32 µg, 0.11 µmol, 0.007 eq) in DOX/H_2_O (6/4, *v*/*v*) (50 µL) was added, and an NMR sample was taken for referencing. The solution was deoxygenated by N_2_-bubbling for 15 min in an ice bath, shaken at 70 °C for 77 min, and the polymerization was subsequently quenched by ice cooling and exposure to air. An NMR sample was taken for conversion determination, and the volatiles were removed at 30 °C under reduced pressure. The residue was dissolved in acetone (3 mL) and the polymer precipitated in ice-cold *n*-hexane (60 mL). This procedure was repeated a further three times until the block copolymer was obtained as a slightly yellow powder, which was dried in vacuo at room temperature for 24 h. Monomer conversion = 33%. SEC: ${\bar{M}}_{n,\mathrm{app}}$ = 14 kDa (${\bar{M}}_{n,\mathrm{th}}$ = 22 kDa), *Ð* = 1.27. For further experimental data, see Table S1; for further analytical data, see Table 2.

#### 2.1.3. Emulsion Polymerizations

The RAFT emulsion polymerizations were conducted in 10-mL screw capped vials sealed with bored poly(propylene) caps and natural rubber/TEF septums. A typical synthesis of PAPy-*b*-PS via surfactant-free aqueous RAFT emulsion polymerization was performed as follows: The PAPy macroRAFT agent/macro-stabilizer (20 mg, 1.1 µmol, 1.0 eq) was dissolved in H_2_O/DOX (8/2, *v*/*v*) (1.0 mL) overnight at 4 °C, and ACVA (62 µg, 0.22 µmol, 0.2 eq) in H_2_O/DOX (8/2, *v*/*v*) (50 µL), as well as styrene (91 mg, 874 µmol, 795 eq) were added (total solids content in the formulation ≈ 10 wt %). The heterogeneous mixture was deoxygenated by N_2_-bubbling for 15 min, homogenized by stirring at 600 rpm and room temperature for 15 min and subsequently polymerized for 5 h at 300 rpm and 70 °C. After ca. 2 h, a pronounced opalescence evolved. The polymerization was quenched by ice cooling and exposure to air, and an NMR sample was taken. For investigation of the nano-objects, a small sample of the latex (100 µL) was diluted with H_2_O (10 mL). The rest of the latex was concentrated under reduced pressure, the polymer redissolved in THF (2 mL) and precipitated in ice-cold *n*-hexane (40 mL). The block copolymer was obtained as a fuzzy colorless solid being dried in vacuo at room temperature for 24 h. Monomer conversion = 98%. SEC: ${\bar{M}}_{n,\mathrm{app}}$ = 77 kDa (${\bar{M}}_{n,\mathrm{th}}$ = 99 kDa, calculated from ${\bar{M}}_{n,\mathrm{th}}$ of the macroRAFT agent), *Ð* = 1.38. For further analytical data, see Table 1 and Table 2, Figure S1c,d.

The syntheses of the PDMA-*b*-P(APi-*co*-APy)-*b*-PS block copolymers were conducted accordingly. For those, it is essential to maintain a constant cooling until the start of the homogenization to avoid undesired early micellization if the PTT of the P(APi-*co*-APy) block is below room temperature.

### 2.2. Analytics

#### 2.2.1. NMR

For the determination of the monomer conversion in the RAFT polymerizations, ^1^H NMR spectroscopy was used. The NMR experiments were conducted on a Bruker Avance II 400-MHz spectrometer (Bruker, Billerica, MA, USA). For a typical ^1^H NMR spectrum, 16 scans were recorded, and a relaxation delay of 3 s was applied. The concentrations were approximately 20 g·L^−1^, and the residual solvent signals were used as the internal reference for the chemical shifts. To estimate the monomer conversions in the syntheses of the PDMA (in CDCl_3_), PAPy and P(APi-*co*-APy) macroRAFT agents (both in D_2_O), respectively, a certain amount of DMF was added to the polymerization mixtures as the internal standard (DMF/acrylamide ≈ 1/6, *v*/*w*). The conversions were then estimated by comparing the DMF/monomer integral ratio before and after polymerization (see Figure S1a). The styrene conversion in the emulsion polymerization steps (determined in THF-d_8_) was calculated from the integral ratio of the aromatic PS signal at 6.73–6.17 ppm (corrected by subtraction of the monomer integral at 6.61 ppm) and the monomer signal at 5.65 ppm.

#### 2.2.2. Size-Exclusion Chromatography (SEC)

SEC was conducted on a PSS Agilent Technologies 1260 Infinity system (PSS, Mainz, Germany; Agilent, Santa Clara, CA, USA) consisting of a precolumn (8 mm × 50 mm) and three analytical columns (8 mm × 300 mm) with a polyester copolymer network (GRAM) as the stationary phase (mesh size 1 × 30 Å and 2 × 1000 Å), a SECcurity auto injector (PSS) and an isocratic SECcurity pump (PSS). The system was operating with the software WinGPC, a refractive index and a UV–Vis detector working at a wave length of 280 nm. As eluent, *N,N*-dimethylacetamide (DMAc) (HPLC Optigrade, Promochem (Wesel, Germany)) with 0.1 M LiCl at a flow rate of 0.8 mL·min^−1^ and a temperature of 50 °C was utilized, and the run time was 60 min. Methyl benzoate was added as the internal standard to the analyzed polymer solutions, which had concentrations of 2–3 g·L^−1^. The sample injection volume was 100 µL. For the determination of apparent molecular weights and *Ð*-values, the system was calibrated with narrowly distributed PMMA (for the polyacrylamide macroRAFT agents) and PS standards (for the styrenic block copolymers).

#### 2.2.3. Visual Turbidimetry

For a quick estimation of cloud points, visual turbidimetry was used. The polymer was dissolved in water and the specific solvent mixture, respectively, at the desired concentration by shaking overnight at 4 °C. The cloud points were determined in three heating-cooling cycles with a reproducibility of <1 °C deviation. The heating was performed in a water bath with a heating rate of about 2 °C·min^−1^. The cooling step was performed at room temperature. The cloud point in our setup was defined as the mean onset of the clouding. The temperature measurements were conducted directly in the sample solution with a Voltcraft PL-120-T1 thermometer (Conrad Electronic AG, Wollerau, Switzerland) using a silver thermostat with the fastest available response rate and a temperature accuracy of 0.1 °C.

#### 2.2.4. Sample Preparation for the Investigation of the Nano-Objects by DLS and CryoTEM

The obtained nano-objects were investigated by DLS and cryoTEM. For that, the samples were directly withdrawn from the raw latexes after polymerization and diluted with the 100-fold excess of water (if not mentioned otherwise) to obtain a final polymer concentration of ca. 1 g·L^−1^ to avoid multiple scattering. The solvents used for dilution were filtered through microporous regenerated cellulose filters (average pore diameter = 200 nm) prior to use.

#### 2.2.5. DLS

The DLS measurements were conducted on an ALV/CGS-3 Compact Goniometer-System (ALV, Langen, Germany) using an ALV/LSE-5003 Multiple Tau Digital Correlator working with pseudo-cross-correlation and the ALV Digital Correlator Software 3.0 (ALV). The measuring angle was set to 90° for all measurements, and every single measurement was conducted for 30 s. As the light source, a Nd:YAG laser emitting at 532 nm was used. The sample vials consisted of quartz glass and were placed into a measurement cell filled with toluene. The temperature-dependent viscosity and refractive index of the solvents were automatically corrected according to tabulated values [49].

Temperature-dependent DLS measurements were conducted in temperature steps of 2 °C with one measurement per temperature. The toluene bath and therefore the samples were tempered by a Julabo F25 thermostat working with a mixture of water and ethylene glycol and delivering a temperature accuracy of 0.01 °C. Each set temperature was stabilized for 3 min prior to measurement. The heating rate was quite slow with ca. 2 °C·h^−1^. The diffusion coefficient *D* (*D* = $\bar{\Gamma }$/*q*^2^) of the particles was calculated automatically by the DLS software from the wave vector *q* and the averaged relaxation rate $\bar{\Gamma }$ by fitting the field autocorrelation function *g*^1^(*q*,*t*) with a cumulant up to second order:

(1) $$\ln\left(g^{1}\left(q,t\right)\right)=\ln A-\bar{\Gamma }\times t+\frac{\mu_{2}}{2}\times t^{2}$$

*t*: time; *A*: amplitude. The *PSD*-values were calculated from the second moment µ_2_ by $PSD=\frac{\mu_{2}}{{\bar{\Gamma }}^{2}}$ and can be understood as the square of the full width at half maximum/mean value of the distribution function.

The *R*_h_-values were estimated from *D* via the Stokes–Einstein equation. Particle size distributions were obtained by fitting the intensity autocorrelation function with a CONTIN algorithm and are depicted as intensity-weighted.

#### 2.2.6. CryoTEM

TEM images were recorded with an Eagle 4k HS 200-kV camera (FEI, Hillsboro, OR, USA) on a FEI Tecnai G2 Spirit TWIN instrument (FEI) in bright field mode, operating at an accelerating voltage of 120 kV. Images were processed with the TEM Imaging & Analysis Offline 4.7 SP3 (FEI) software and ImageJ 1.51p. Samples were prepared on carbon-coated copper grids or lacey carbon grids (Quantifoil, Großlöbichau, Germany) using a Vitrobot Mark IV (FEI) at 100% humidity and temperatures in between 4 and 8 °C, if not mentioned otherwise. The latexes were dropcast onto the TEM grid before the sample excess was blotted with filter paper for 2 s. Samples prepared at higher temperatures were left equilibrating for 10 s prior to blotting. The blotted grids were allowed to rest for 1 s, then vitrified by rapid immersion in liquid ethane and stored in liquid nitrogen until measurement.

## 3. Results

### 3.1. Preliminary Remarks and Solubility Tests

**Figure 2:**
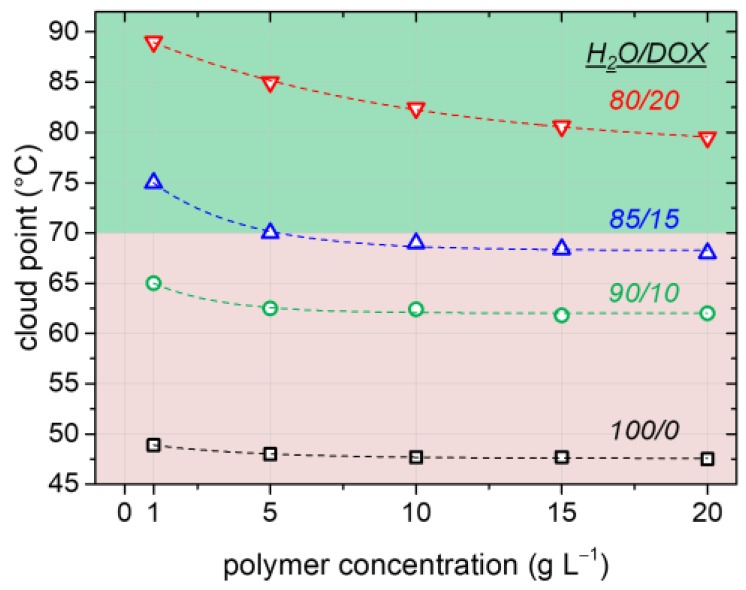
Cloud point versus polymer concentration of PAPy in different H_2_O/DOX mixtures indicated above the related curve (in *v*/*v*). The common polymer concentration used for the emulsion polymerizations is 20 g·L^−1^. If the cloud point of the thermoresponsive macroRAFT agent/macro-stabilizer is below the polymerization temperature, no emulsion polymerization is possible (red region); if it is above, the latex can efficiently be stabilized (green region).

For the surfactant-free RAFT emulsion polymerization, a well-dissolved macroRAFT agent/macro-stabilizer is vital to stabilize the formed latex and prevent coagulation. This, obviously, lets thermoresponsive LCST-type macroRAFT agents a priori appear unsuitable as they are usually insoluble at the common polymerization temperatures of 65–70 °C. To still be able to use the tremendous advantages of RAFT emulsion techniques for synthesizing high-molecular-weight thermoresponsive styrenic block copolymers, we make use of adding slight amounts of a cosolvent pushing the PTT of the used PAPy macroRAFT agents upwards and hence making them soluble in the polymerization medium at 70 °C (Figure 2). It is, however, essential to be aware of cononsolvency effects, which can appear when certain organic cosolvents are added to aqueous polymer solutions [46,50,51,52]. This effect leads to a lower solubility of the polymer at low additive amounts (that is, the PTT decreases) and is most often observed for aliphatic alcohols as additives. The careful choice of the cosolvent is therefore crucial for achieving the desired solubility enhancement.

**Figure 3:**
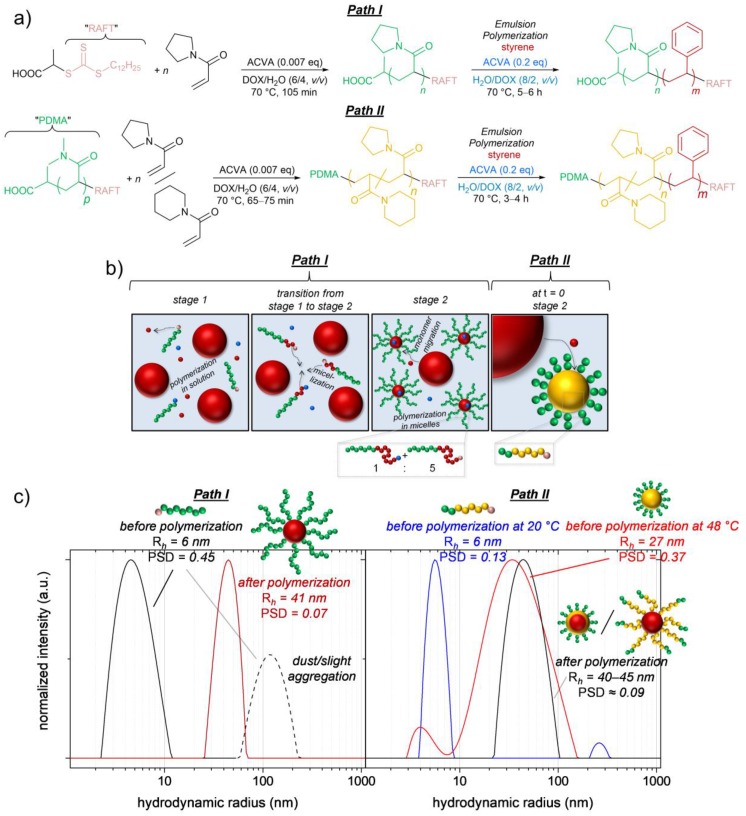
Synthetic pathways to the targeted thermoresponsive styrenic block copolymers. (**a**) Path I: Synthesis of PAPy-*b*-PS via emulsion polymerization. Path II: Synthesis of PDMA-*b*-P(APi-*co*-APy)-*b*-PS via emulsion polymerization using a nanoreactor approach [41]; (**b**) Sketched mechanism for the emulsion polymerizations. The colors used fit the ones used for the respective components in the reaction schemes in (a). The third mechanistic stage in which the monomer droplets are fully consumed is not drawn; (**c**) Particle size distributions (obtained via CONTIN analysis), hydrodynamic radii (*R*_h_) and particle size dispersities (*PSD*) (both obtained via cumulant fitting) of the initial polymer/solvent systems and of the final latexes (in this particular case, determined in H_2_O/DOX (8/2, *v*/*v*)). In Path I, the initial state is a dissolved PAPy random coil, and the final latex contains PAPy-*b*-PS micelles. In Path II (exemplarily shown for the synthesis of D_2_(I_70_Y_30_)_19_S_79_^103^), the initial state is dissolved PDMA-*b*-P(APi-*co*-APy) coils at temperatures below the phase transition temperature (PTT) of the random block and P(APi-*co*-APy)-core micelles at higher temperatures. The final latex contains PS-core micelles with a PDMA outer corona and a P(APi-*co*-APy) shell in the collapsed or coiled state, respectively, depending on the temperature (the shown DLS data for this latex are obtained in pure H_2_O and should hence be compared only qualitatively with the other samples).

Figure 2 depicts that the addition of low amounts of DOX as the cosolvent indeed increases the cloud point of PAPy significantly from 47 °C in pure H_2_O to ca. 80 °C in the presence of 20 vol % DOX. Besides its positive effect on the solubility of the used PAPy macroRAFT agents, the added DOX furthermore slightly increases the solubility of styrene in the aqueous phase (we experimentally evaluated the styrene concentration in the H_2_O/DOX phase to be roughly 4–5 mM at room temperature compared to a reported concentration of ca. 3 mM in pure water [53]). While the still low styrene solubility in the continuous phase leads to the maintenance of a true emulsion polymerization mechanism, the slightly increased styrene concentration can be beneficial for faster kinetics of the initial chain extension of the solvophilic macroRAFT agent before micellization (Figure 3b, Stage 1). This is due to the fact that the kinetics in this stage resembles the ones of a common RAFT solution polymerization with very low monomer concentration; hence, the polymerization rate is here proportionally increasing with an increasing monomer concentration. As will be shown in the following, the addition of DOX moreover has no negative influence on the stability of the latex (i.e., no significant amount of coagulum appears; see Figure S3) or on the control and kinetics of the polymerization due to enhanced radical exiting [54,55]. The addition of a cosolvent thus seems to be a versatile tool to solubilize thermoresponsive macroRAFT agents and stabilizers, at least if their PTTs are sufficiently high.

The described cosolvent approach can be additionally combined with the attachment of a stabilizing hydrophilic non-thermoresponsive block (in our case PDMA) to the thermoresponsive macroRAFT agent (Figure 3, Path II). This approach is similar to the above-mentioned nanoreactor approach by Monteiro et al., but we used a significantly shorter, though still effectively stabilizing, PDMA block to not differ too much from our targeted thermoresponsive block copolymer structure (PDMA/P(APi-*co*-APy) ≈ 1/10 (*w*/*w*) in our case compared to PDMA/PNIPAm ≈ 1/2 [40] and 1/1 [41,42], respectively, presented by Monteiro et al.). Actually, the main advantage of this approach is its universality and transformability to other thermoresponsive systems with different PTTs, making a further adjustment of the macroRAFT structure or of the solvent mixture unnecessary. This is even valid in case the main thermoresponsive component of the macroRAFT agent is insoluble in the aqueous phase at the polymerization temperature. We for instance adjusted the aqueous PTT of the P(APi-*co*-APy) block in between 4 and 47 °C (PTT ≈ 7–80 °C in the polymerization medium H_2_O/DOX (8/2, *v*/*v*)) by varying its APi/APy ratio (the higher the APy content, the higher the PTT) [46], and the latexes formed in the emulsion polymerization process were fully stable in all cases. It should be mentioned here, however, that the self-assembly in these polymerizations is induced by the temperature-induced collapse of the thermoresponsive block rather than by PISA in most cases (where PTT < 70 °C, Figure 3b,c). Due to this pre-polymerization micellization, Stage 1 in the emulsion polymerization mechanism is skipped, and a fast polymerization inside the micelles takes place from the beginning of the heating (polymerization times to styrene conversions of >90% reduce from 5–6 h in the emulsion polymerizations using the PAPy macroRAFT agents to 3–4 h in the emulsion polymerizations using PDMA-*b*-P(APi-*co*-APy) macroRAFT agents).

### 3.2. Kinetics of the Synthesis of PAPy-b-PS via RAFT Emulsion Polymerization

**Figure 4:**
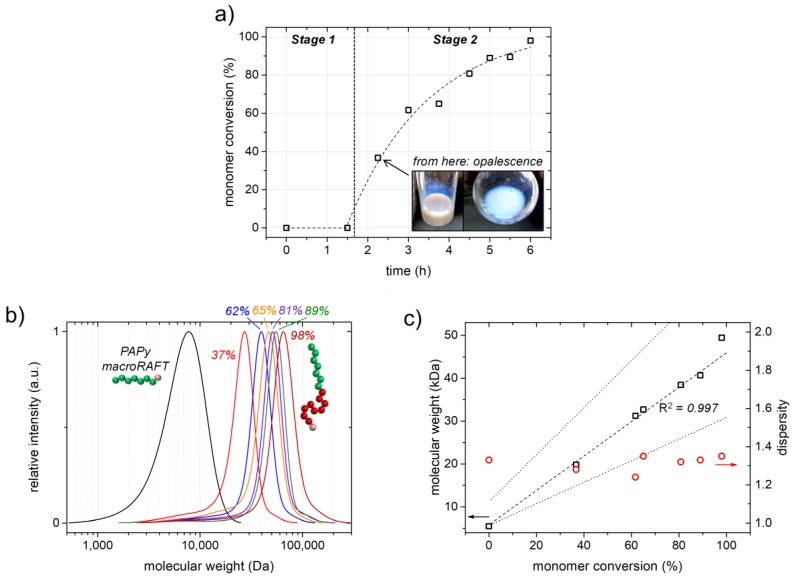
Kinetic investigation of the RAFT emulsion polymerization of styrene using the PAPy macroRAFT agent/macro-stabilizer Y^12^ (see Table 1 and Figure 3, Path I). (**a**) Styrene conversion versus time. The indicated mechanistic stages correspond to Figure 3b, Path I. The fit is supposed to guide the eye; (**b**) Evolution of the SEC traces with monomer conversion (values indicated in the respective color) determined by DMAc-SEC; (**c**) Apparent number-average molecular weights (${\bar{M}}_{n,\mathrm{app}}$) (left ordinate, black squares) with linear fit (dashed line) and the corresponding regression coefficient *R*^2^, as well as molecular weight dispersities (right ordinate, red circles) versus styrene conversion. The lower dotted line indicates the evolution of ${\bar{M}}_{n,\mathrm{th}}$ calculated with ${\bar{M}}_{n,\mathrm{app}}$ of the PAPy macroRAFT agent; the upper one indicates ${\bar{M}}_{n,\mathrm{th}}$ calculated with ${\bar{M}}_{n,\mathrm{th}}$ of the PAPy macroRAFT agent (for the exact formula, see footer of Table 1).

The PAPy used as both macroRAFT agent and macro-stabilizer in the emulsion polymerization of styrene (Figure 3, Path I) was synthesized with a very high theoretical livingness according to synthetic principles published by Perrier et al. [56,57,58] as is described elsewhere in more detail [47]. In brief, the monomer APy was polymerized to conversions of ca. 50% in DOX with the cosolvent H_2_O (6/4, *v*/*v*) using the commercially available RAFT agent DTPA and very low amounts of the initiator ACVA ([DTPA]/[ACVA] = 150/1). The apparent number-average molecular weights (${\bar{M}}_{n,\mathrm{app}}$) of PAPy (obtained by DMAc-SEC using PMMA standards) are usually significantly lower than the theoretical ones (${\bar{M}}_{n,\mathrm{th}}$) by about 30–50%, and the molecular weight dispersities (*Đ*) are moderate with *Đ* = 1.25–1.35. This rather high discrepancy between ${\bar{M}}_{n,\mathrm{th}}$ and ${\bar{M}}_{n,\mathrm{app}}$ probably goes back to interactions of the polar PAPy with the GRAM solid phase of the SEC column and to a different hydrodynamic volume of sample and standard. We thus assume that the ‘true’ ${\bar{M}}_{n}$ of the PAPy macroRAFT agents is somewhere in between ${\bar{M}}_{n,\mathrm{th}}$ and ${\bar{M}}_{n,\mathrm{app}}$ (see also Figure 4c); ${\bar{M}}_{n,\mathrm{th}}$, anyway, is used in their sample codes (Figure 1).

By dissolving the PAPy macroRAFT agent (in this case sample Y^12^, Table 1) in H_2_O/DOX (8/2, *v*/*v*), adding ACVA as initiator ([PAPy macroRAFT]/[ACVA] = 5/1) and the monomer styrene, the emulsion polymerization formulation was created. If not mentioned otherwise, we adjusted the systems to solvent/styrene/PAPy = 100/9/2 (*w*/*w*/*w*) so that the PS weight fraction in the generated block copolymers will be ca. 80 wt % at high styrene conversions.

The kinetic investigation of the RAFT emulsion polymerization depicted in Figure 4 reveals an initial period with only very low styrene conversion extending over 1.5 h after which the polymerization rate increases dramatically and an almost quantitative styrene conversion is achieved within the following 3–4 h. This dramatic change of polymerization kinetics is caused by the mechanistic transition of the system from macroRAFT chain extension in the aqueous phase (Stage 1) to polymerization inside the block copolymer micelles (Stage 2) being formed by micellization at a critical PS block length (Figure 3b). Macroscopically, this process can be nicely observed by the development of a pronounced opalescence of the sample due to Mie scattering (the so-called Tyndall effect; see the inset of Figure 4a).

The SEC traces of the obtained PAPy-*b*-PS block copolymers clearly shift to higher molecular weights upon styrene conversion with no or only very little low-molecular-weight tailing, which indicates a high livingness of the PAPy macroRAFT agent, as well as a high blocking efficiency in the emulsion polymerization (Figure 4b and Figure S2). This is also valid for low styrene conversions suggesting an almost simultaneous micellization and micelle nucleation in the whole system and a high control of the PAPy chain extension with styrene in the aqueous phase. If this were not the case, a fraction of unextended macroRAFT agents would appear in the SEC measurements of the block copolymers obtained at low conversions, as has been reported for some other emulsion polymerizations [8,20,59]. A high control in our RAFT emulsion polymerization system is furthermore indicated by a linear increase of the block copolymers’ molecular weights with styrene conversion, as well as by low and constant *Đ*-values (*Đ* = 1.25–1.35) (Figure 4c and Figure S2).

Since polyacrylamide macroRAFT agents are usually bad chain transfer agents for more activated monomers (MAMs) like styrene in common homogeneous RAFT polymerizations [1,60], the possibility to synthesize well-controlled polyacrylamide-*b*-PS block copolymers actually goes back to the beneficial and exclusive properties of emulsion polymerizations: During the chain extension of the PAPy macroRAFT agents with the first few styrene units in Stage 1 of the emulsion polymerization mechanism, the styrene concentration is low and almost constant. This favors the chain transfer reaction from the oligomeric PS radicals to the dormant PAPy chains over chain propagation and thus promotes a simultaneous chain extension [60,61,62,63]. After micellization, the styrene concentration and propagation rate increase tremendously, though this has no negative influence on the polymerization control anymore because the growing radicals are now of styrenic nature.

To conclude, by the presented cosolvent approach, thermoresponsive block copolymers with a major PS block can be synthesized with high control in short polymerization times.

### 3.3. Synthesis and Self-Assembly of PAPy-b-PS with Different PS Block Lengths

To achieve higher molecular weights of the block copolymers in the emulsion polymerizations, we increased the chain length of the used PAPy macroRAFT agent/macro-stabilizer. In the following, the macroRAFT agent Y^18^ is used (${\bar{M}}_{n,\mathrm{app}}$ = 12 kDa, Table 1). While keeping the styrene amount in the emulsion formulations constant at a value of ca. 10 wt %, we varied the amount of the macroRAFT agent to generate block copolymers with different block ratios (68–93 wt % PS) and molecular weights (${\bar{M}}_{n,\mathrm{th}}$ = 60–300 kDa), as well as to check for the upper molecular weight limit we can approach with our emulsion system in a controlled manner.

**Figure 5:**
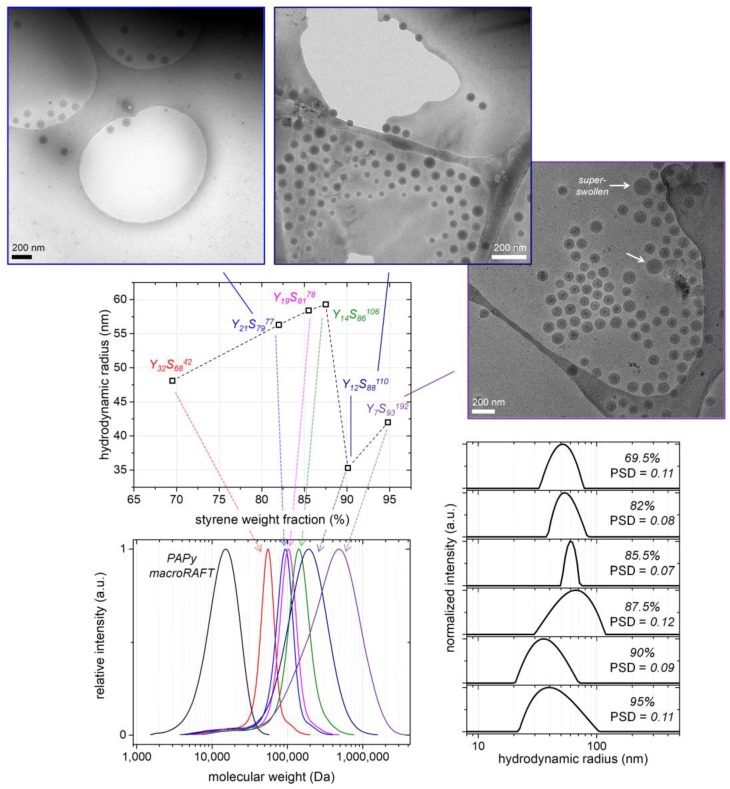
Synthesis of PAPy-*b*-PS with different PS block lengths using Y^18^ as the PAPy macroRAFT agent (see also Figure 3, Path I, and Table 1). Shown are results for the final latexes after the emulsion polymerization. Hydrodynamic micelle radius (obtained by DLS and cumulant fitting) versus styrene weight fraction used in the emulsion polymerizations (compared to PAPy macroRAFT agent), the sample codes (see Table 1) are indicated. Three cryoTEM images are exemplarily shown (for more images; see Figures S6–S9). In the image of sample Y_7_S_93_^192^ (95% styrene weight fraction), two “unsplit” micelles are indicated, potentially leading to a molecular weight broadening of the block copolymer. Furthermore, the related SEC traces of the block copolymers (colors correspond to the sample codes), as well as particle size distributions (obtained by CONTIN analysis, styrene weight fractions are indicated) with related size dispersities (*PSD*) (obtained by cumulant fitting) are given.

Indeed, the amount of coagulum which formed in the emulsion polymerizations was quite low in all cases (ca. 1–2 wt %; see Figure S3), suggesting a stable latex even in case a very high excess of styrene compared to the PAPy macroRAFT agent is used. Hence, the latter proves to be an efficient macro-stabilizer under the investigated experimental conditions. Figure 5 and Table 1 moreover indicate that the control of the polymerization is high up to PS block fractions of 86 wt %, thus up to ${\bar{M}}_{n,\mathrm{th}}$ ≈ 140 kDa (${\bar{M}}_{n,\mathrm{app}}$ ≈ 110 kDa); that is to say, the *Đ*-values of the block copolymers are below 1.50. Above that limit, the molecular weight distributions of the block copolymers broaden significantly.

By using DLS and cryoTEM, the PAPy-*b*-PS nano-objects in solution and hence the PISA simultaneously taking place with the PS chain extension are investigated; the results are depicted in Figure 5. The investigations were performed in water as was usually done in this work if not mentioned otherwise (samples obtained from the emulsion polymerizations were diluted with the 100-fold excess of water; see Materials and Methods. Concerning the cryoTEM images, it should additionally be noted that only the dense PS core of the micelles can be seen because of the low contrast of the swollen corona in regard to water.

Anyway, it can be stated that all PISA-generated PS-core-PAPy-shell micelles are spherical and well-defined with low particle size dispersities (*PSD*, for its definition see Materials and Methods ) in between 0.07 and 0.12 determined by DLS. The hydrodynamic radius (*R*_h_) of the micelles first increases with the styrene fraction from 48 nm at 69.5 wt % styrene (compared to 30.5 wt % PAPy macroRAFT) to 59 nm at a styrene fraction of 87.5 wt %, which can be attributed to an increasing core size. However, the aggregation number of the micelles (*N*_agg_) is probably constant in those cases as they are formed in the early stages of the polymerization when the PS block is very short, and the chains become somewhat locked up upon further PS block extension as a consequence of the negligible chain diffusion through the continuous aqueous phase. When the styrene amount per micelle and hence the swelling becomes higher with further styrene addition, though, the micelles split up (probably induced by shear) and thus decrease in size. This critical swelling degree is reached at roughly 87.5 wt % styrene and can be observed as a micelle *R*_h_-drop by 25 nm; the particle size distribution curve of sample Y_14_S_86_^106^ (styrene fraction of 87.5 wt %; Table 1) indeed already shows a broadening to lower *R*_h_-values (Figure 5). Decreasing particle sizes at very low stabilizer concentrations have been reported by other groups, as well [39]. This phenomenon might be related to a superswelling of the micelles formed early in Stage 1 of the polymerization process [39,59,64]. Since the macroRAFT and thus radical concentration in the systems with high styrene fractions is very low, the (super)swelling of the micelles might be much faster than their nucleation and the growth of the PS block (which locks the micelle structure at some point). Therefore, the micelles retain a soft and mechanically labile structure over extended time periods, facilitating the shear-induced breakup and hence size decrease.

Noticeably, the block copolymers being obtained at styrene fractions above the critical value own a broader molecular weight distribution indicating a lower degree of polymerization control, which might be partially due to the very low concentration of RAFT agent in the system (possibly there is a higher contribution of zero–one compared to pseudo-bulk kinetics) [54,55]. While the *PSD*-values of the related micelles, nevertheless, are low (*PSD* = 0.09–0.11) showing that the PISA process is still mostly well-defined, the cryoTEM images reveal a small fraction of large ‘unsplit’ micelles (Figure 5 and Figure S9). The participation of that fraction of larger micelles in the polymerization process might be another reason for the rather broad molecular weight distributions of those samples. As can furthermore be seen in the cryoTEM images, different micelle morphologies than spherical, such as worm-like micelles or vesicles, do not appear even at these high styrene fractions at which the morphological transformation should be thermodynamically favored due to the packing parameter. This is a consequence of the rather low total solids content in the formulations of 10 wt % and the rather long stabilizing PAPy block hindering the initially spherical micelles to fuse and by that form cylindrical structures, as well as due to the long core-forming PS block hindering the chains from diffusion [23,25]. Tackling these reduced dynamics, Truong and Davis et al. [43], as well as Monteiro et al. [35] presented that they can be circumvented by adding toluene as a plasticizer for the PS block (see also the Introduction), which is not part of this work, though.

As a summary, high-molecular-weight narrowly size-distributed thermoresponsive block copolymers are obtainable by the presented emulsion polymerization approach. While above a critical level of styrene fraction, however, the molecular weight control by the RAFT process decreases, the micelles generated via PISA own low *PSD*-values even in case of very high styrene fractions (i.e., low PAPy block fractions). Hence, well-defined spherical star-like, as well as crew-cut micelles with a glassy PS core and a thermoresponsive PAPy corona can be generated in a straightforward manner.

### 3.4. Synthesis and Self-Assembly of PDMA-b-P(APi-co-APy)-b-PS with Different Monomer Ratios in the Random Block

**Figure 6:**
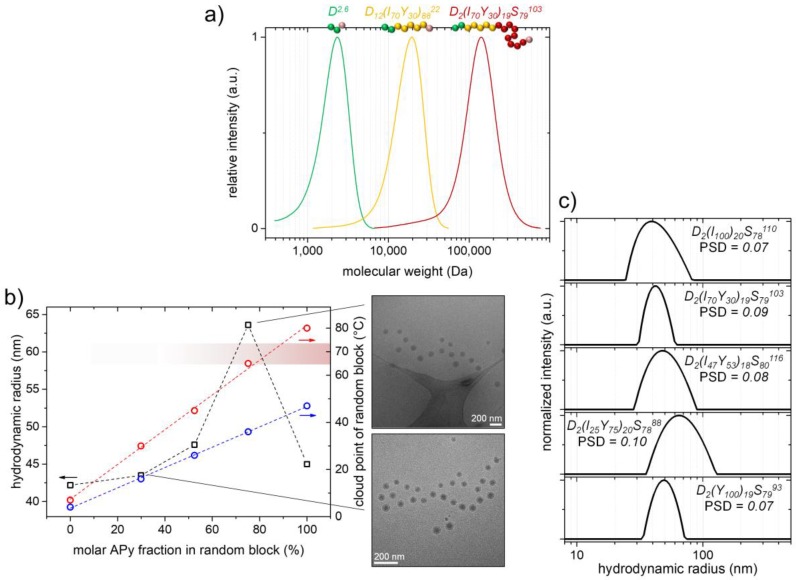
Synthesis and self-assembly of PDMA-*b*-P(APi-*co*-APy)-*b*-PS obtained by the nanoreactor approach. (**a**) SEC traces of the PDMA and PDMA-*b*-P(APi-*co*-APy) macroRAFT agent, as well as of sample D_2_(I_70_Y_30_)_19_S_79_^103^ (see also Figure S4); (**b**) Micellar *R*_h_-values in water (left ordinate, black squares), cloud point of the P(APi-*co*-APy) block in H_2_O/DOX (8/2, *v*/*v*) (right ordinate, red circles, approximately determined by visual turbidimetry) and in H_2_O (right ordinate, blue circles, theoretically calculated by PTT_th_ = *x*_APi_ × 4 °C + *x*_APy_ × 47 °C [46,47]) versus the molar APy fraction in that random block. The polymerization temperature region is indicated as a red band on the right. The *R*_h_-values were measured at 10 °C below PTT_th_ in water, except for sample D_2_(I_100_)_20_S_78_^110^, which was measured at 5 °C. Additionally, cryoTEM images of the samples D_2_(I_70_Y_30_)_19_S_79_^103^ and D_2_(I_25_Y_75_)_20_S_78_^88^ are exemplarily shown (see Figures S10 and S11 for more images); (**c**) Particle size distributions for the different triblock copolymer micelles in water (increasing APy fraction from top to bottom) and the related size dispersity (*PSD*).

As has already been pointed out in the above discussion of the preliminary investigations, we furthermore combined the just described cosolvent approach with the nanoreactor approach presented by Monteiro et al. (Figure 3, Path II) [40,41]. According to that, we used a short temperature-insensitive and very hydrophilic PDMA block (${\bar{M}}_{n,\mathrm{th}}$ = 2.6 kDa, ${\bar{M}}_{n,\mathrm{app}}$ = 1.7 kDa) synthesized by RAFT polymerization as a stabilizer and attached a thermoresponsive P(APi-*co*-APy) random copolymer block with a widely and linearly adjustable PTT to it (Figure 6b). The synthetic conditions used to obtain the block copolymeric macroRAFT agents were similar to the ones utilized for synthesizing the PAPy macroRAFT agents (i.e., we used very low amounts of the initiator and DOX/H_2_O (6/4, *v*/*v*) as solvent). Well-defined polymers with the desired molecular weights (${\bar{M}}_{n,\mathrm{th}}$ = 22–28 kDa, ${\bar{M}}_{n,\mathrm{app}}$ = 14–18 kDa), as well as with low *Đ*-values (*Đ* < 1.30 except for D_11_(Y_100_)_89_^24^ with *Đ* = 1.45 probably due to stronger column interactions) were obtained (Table 2).

In case the PTT of the thermoresponsive block is below the polymerization temperature of 70 °C, which is the case for a molar APy content below ca. 75% in the random block, the macroRAFT agents self-assemble into micelles already from the start of the emulsion polymerization. Hence, they swell almost immediately with styrene and the growth of the PS block proceeds quickly inside the solvophobic micelle core (Figure 3, Path II). The characteristic opalescence of the latexes indicating the beginning chain growth appears already after a few minutes of heating in those cases. With that being the case, the macroRAFT agents D_9_(I_100_)_91_^28^, D_12_(I_70_Y_30_)_88_^22^ and D_10_(I_47_Y_53_)_90_^26^ in fact allow for a very fast RAFT emulsion polymerization delivering quantitative styrene conversions within ca. 3 h polymerization time and triblock copolymer molecular weights above 100 kDa (we again aimed for block copolymers with ca. 80 wt % PS fraction; Table 2). Regarding these high molecular weights, the *Đ*-values of the triblock copolymers in between 1.36 and 1.64 are satisfying. The slightly higher *Đ*-values compared to the PAPy-*b*-PS samples go back to the different polymerization mechanism in which also the initial chain extension of the polyacrylamide macroRAFT agents with styrene takes place inside the micelles, i.e., at a high local styrene concentration, and not in the low concentrated continuous phase as in the PAPy macroRAFT systems. Hence, the initial chain transfer from the oligomeric PS radicals to the dormant polyacrylamide chains is less preferred compared to the PS chain propagation, which results in a slight broadening of the molecular weight distributions [60,63]. Nevertheless, what is more important to us is that the SEC results indicate a very high to quantitative blocking efficiency of the macroRAFT agents. That can be deduced from only a very slight or even completely absent fraction of unextended macroRAFT chains appearing in the SEC traces of the triblock copolymers (Figure 6a and Figure S4).

Coming to the macroRAFT agents/macro-stabilizers with higher APy contents and hence PTTs, the macroRAFT agent D_11_(Y_100_)_89_^24^ with its PTT well above the polymerization temperature shows the expected unseeded emulsion polymerization behavior (polymerization time to full conversion ≈ 6 h). On the other hand, the RAFT agent D_9_(I_25_Y_75_)_91_^28^ behaves more complex due to the proximity of its PTT to 70 °C (Figure 6b). The polymerization kinetics in that sample are very sensitive to slight variations in the polymerization temperature and have turned out to be somewhat unpredictable. This is even the case when the polymerization temperature is increased further (to induce a complete collapse of the P(APi-*co*-APy) block) or more DOX as cosolvent is added (to increase the PTT to temperatures well above 70 °C). Our explanations for this stubborn behavior are still elusive; anyway, we think that it might result from the certain composition of solvophilic APy and solvophobic APi units in the random block, which might lead to a higher core solvation of the ab initio micelles [47] hindering the styrene to diffuse in. Furthermore, we could imagine a rather gradient-like collapse of the random block in combination with the hydrophilic PDMA block leading to a different behavior compared to the other samples [65,66].

The generated micelles during polymerization (we try to omit the term ‘PISA’ in this context since the self-assembly is mostly temperature-induced except for sample D_2_(Y_100_)_19_S_79_^93^, as described above) all show narrow particle size distributions with *PSD*-values of 0.07–0.10 (Figure 6c). Except for sample D_2_(I_25_Y_75_)_20_S_78_^88^, their sizes are in the range of *R*_h_ = 42–47 nm. Considering that the coronas of the micelles are thermoresponsive and hence their size is temperature-dependent (see the section below), this size range is quite narrow and the micelles seem to be of a similar *N*_agg_ and morphology (the given *R*_h_-values in Figure 6b are measured in water at temperatures 10 °C below the aqueous PTT_th_ of the P(APi-*co*-APy) block). Compared to the above presented micelles of the PAPy-*b*-PS samples, however, the triblock copolymer micelles are significantly smaller, even in case of the non-ab initio system of D_2_(Y_100_)_19_S_79_^93^. This indicates a lower *N*_agg_ of the latter due to the longer corona blocks and the strongly hydrated, hence, bulky PDMA end block leading to larger coil dimensions and, therefore, to a stronger corona chain repulsion [67].

Sample D_2_(I_25_Y_75_)_20_S_78_^88^ appears once more somewhat special as the *R*_h_-value of its micelles is by about 20 nm (≙50%) higher than the *R*_h_-values of the other four samples. Potentially, the high core solvation of the ab initio formed D_9_(I_25_Y_75_)_91_^28^ micelles being caused by the high number of solvophilic APy units in the core leads to a looser packing and, as a result, to a higher *N*_agg_ of the micelles. As already mentioned above, it could moreover be hypothesized that the micelles own rather gradient-like properties (“reel-in” effects, etc.) and hence show a different behavior compared to the other samples behaving rather block-like [66].

Anyway, the combined emulsion polymerization-nanoreactor approach presented in this section proves feasible for synthesizing styrenic block copolymers with widely selectable thermoresponsive blocks and molecular weights above 100 kDa in a controlled and fast fashion. Self-assembled well-defined micelles are additionally provided by the emulsion polymerization process. Furthermore important, the minimal fraction of the stabilizing PDMA block does not significantly influence the desired block copolymer properties, as will also be pointed out in the following section.

### 3.5. Thermoresponsiveness of the Created Nano-Objects

In the latter sections, we have shown that micelles with narrow particle size distributions are generated by self-assembly in both presented synthetic paths, either polymerization-induced or temperature-induced before polymerization. What can be questioned up to here, especially for the micelles obtained in the nanoreactor approach, is, however, whether the thermoresponsive P(APi-*co*-APy) block is highly entangled and therefore buried inside the micelle core due to its solvophobicity at the polymerization temperature or rather forms a second inner shell besides the outer hydrophilic PDMA corona (as it is sketched in Figure 3c). In the former case, no thermoresponsiveness of the micelles would be expected, while in the latter case, the thermoresponsiveness of the middle block could be addressed.

**Figure 7:**
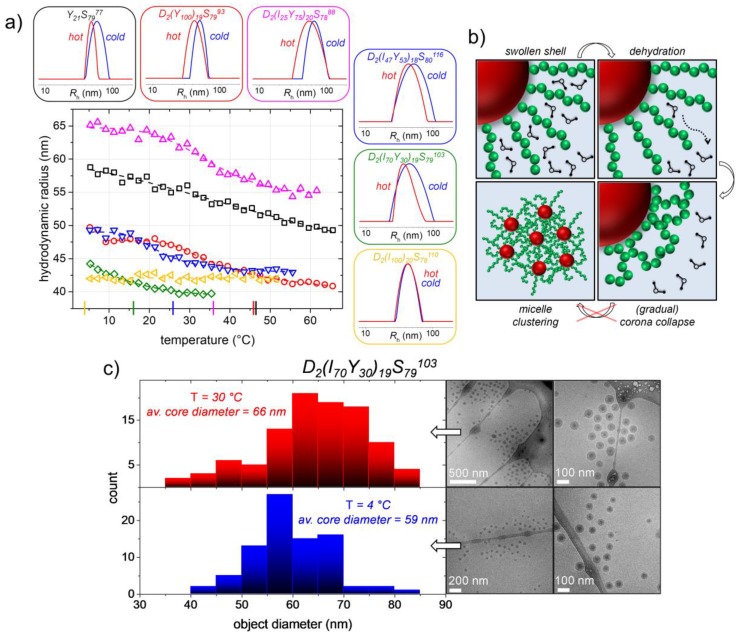
Investigation of the thermoresponsiveness of the micelles generated in the emulsion polymerizations. (**a**) Hydrodynamic radius versus temperature obtained by temperature-dependent DLS and cumulant fitting. The values for PTT_th_ of the PAPy and P(APi-*co*-APy) blocks, respectively, are indicated on the *x*-axis in the respective color. Additionally, representative particle size distributions at low and high temperatures determined by a CONTIN analysis are given; (**b**) Schematic mechanism for the thermoresponsiveness of the micelles. As indicated, a micelle clustering after corona collapse does not take place due to the missing chance for intermicellar entanglements; (**c**) Temperature-dependent cryoTEM images and evaluation of the particle sizes of sample D_2_(I_70_Y_30_)_19_S_79_^103^ (see Figure S11 for more images). Indicated are the Vitrobot temperatures at which the samples were prepared. In the images, only the dense micelle core is visible because of the low contrast of the swollen corona against water. The apparent core size increases by the temperature-induced corona collapse.

Thus, to check for the thermoresponsiveness of the micelles, we used temperature-dependent DLS and looked at the development of the *R*_h_-values and particle size distributions. The results for these investigations of the five different triblock copolymer samples (Table 2), as well as of the diblock copolymer Y_21_S_79_^77^ (Table 1) are depicted in Figure 7.

A temperature-dependent micelle size is observed for all samples except D_2_(I_100_)_20_S_78_^110^, the PTT_th_ of which is at 4 °C and therefore below the accessible temperature in our experimental setup. This polymer is hence in its collapsed coronal state over the whole investigated temperature range. By heating up the other aqueous micellar solutions to temperatures above their PTT, however, the initially hydrated coronal PAPy and P(APi-*co*-APy) block, respectively, becomes dehydrated, collapses and the *R*_h_-value of the micelles decreases (Figure 7). Figure S5 furthermore shows that the investigated temperature-induced corona collapse is fully reversible.

Usually, the decrease in micelle size has a magnitude of 15–18% of the initial size, which fits quite well with the weight fraction of the thermoresponsive block of roughly 20%. In the case of sample D_2_(I_70_Y_30_)_19_S_79_^103^ (PTT_th_ = 16 °C), the *R*_h_-drop is a little less pronounced with roughly 10%. The latter is probably caused by an already slightly collapsed P(APi-*co*-APy) block at the starting temperature of the measurement at 5 °C. Moreover, as was exemplarily done for this sample and as is shown in Figure 7, the temperature-induced corona collapse can be followed by temperature-dependent cryoTEM. In those images, an increase in core radius is observed, which is similar in size to the *R*_h_-decrease measured by DLS (3.5-nm core radius increase compared to 4-nm *R*_h_-decrease). This indicates the formation of an onion-like core structure with an inner PS core wrapped up in a dense P(APi-*co*-APy) shell. All these results suggest that the thermoresponsive blocks are indeed readily accessible for the solvent water, and their thermoresponsiveness can thus be addressed by varying the solution temperature. In fact, this indicates that the core-shell and core-shell-shell micelle structures, respectively, schematically drawn in Figure 3c, are correct.

Although given this apparent thermoresponsiveness of the micelles and the proximity of the PTT to its theoretical value [47], the corona collapse appears to be rather gradual than stepwise, a behavior which is different to their single-block analogues owning a very sharp transition [46]. Sticking to the common vocabulary, the temperature-dependent behavior of the micelles should thus rather be named “thermosensitive” than “thermoresponsive”. We assume that the more gradual corona collapse is a consequence of the high chain density in the corona hindering them from collapsing freely and instantaneously and which additionally leads to a decreased temperature onset of the phase transition in relation to PTT_th_. Furthermore, the absence of bulky side chains in the polyacrylamides reduces the chance of chain entanglements, which would reinforce the thermoresponsive collapse [68].

What can furthermore be noticed is that an intermicellar clustering after collapse of the thermoresponsive blocks does not take place (Figure 7). While, on the one hand, this could be expected for the triblock systems due to the hydrophilic PDMA block stabilizing the latex, it is on the other hand remarkable for sample Y_21_S_79_^77^ lacking this additional shell. We actually observed this behavior also for other micellar systems owning PAPy coronas and suppose that it goes back to the high curvature of the micelles, i.e., the low interfacial area when two micelles approach each other, as well as to the low free volume in the collapsed corona [47]. The latter is indicated by the high bulk-*T*_g_ of the polyacrylamides being in between 116 °C (pure PAPi) and 142 °C (pure PAPy) [47]. Both factors reduce the chance of the micelles to interpenetrate, form intercoronal entanglements and by that micelle clusters; figuratively speaking, the micelles behave like hard glass balls. The missing possibility for intermicellar entanglements above the corona collapse is in fact one major reason for the temperature-reversibility of the system (Figure S5) [68]. Moreover, important for the reversibility is that the low free volume in the collapsed coronas does apparently not significantly inhibit their reswelling with water when the system is recooled down to temperatures below its PTT.

As a result, the micelles generated in the emulsion polymerization processes turn out to be thermosensitive with an adjustable PTT, indeed. At the same time, the micelles are also stable when the thermoresponsive coronal block is in its collapsed state, i.e., at higher temperatures. By the two presented synthetic methods, hence, not only well-defined amphiphilic block copolymers with high molecular weights can be obtained, but also temperature-switchable nano-objects.

## 4. Conclusions

In this work, it has been shown that amphiphilic thermoresponsive block copolymers can be synthesized by surfactant-free RAFT emulsion polymerization in short reaction times. In one part, we have focused on block copolymers with molecular weights of ca. 100 kDa, a major PS block (ca. 80 wt %) and a minor thermoresponsive PAPy block (ca. 20 wt %). The stability of the latex formed by PISA during the emulsion polymerization process, containing spherical micelles with a PS core and a stabilizing PAPy corona, was achieved by adding 20 vol % of the cosolvent DOX to the solvent water. This increases the PTT of the PAPy macroRAFT agent/macro-stabilizer above the polymerization temperature of 70 °C. Furthermore, this method was combined with a nanoreactor approach utilizing a short hydrophilic PDMA block (2 wt % in the final triblock copolymer) as a very efficient and temperature-insensitive stabilizer for the ab initio formed micelles with a thermoresponsive P(APi-*co*-APy) core. The latter continuously grows by chain extension of the block copolymeric macroRAFT agent with styrene. Beneficially in this approach, the PTT of the thermoresponsive random block can be freely adjusted by varying its APi/APy ratio, while the formerly mentioned unseeded RAFT emulsion polymerization proves useful for thermoresponsive macroRAFT agents with a sufficiently high PTT.

Both presented emulsion approaches provide a full chain growth of the PS block within 3–6 h, being dramatically faster than conventional RAFT solution or dispersion polymerizations of styrene. In those types of RAFT polymerizations, ca. 10% styrene conversion per day, maximal conversions of roughly 30–40% and, thus, block copolymer molecular weights of maximal 50–70 kDa are usually achievable under feasible conditions in systems comparable to ours [69]. Moreover, since acrylamides are less activated monomers than styrene, the low reinitiation efficiency of the polyacrylamide macroRAFT agents would prohibit the formation of well-defined block copolymers in a homogeneous RAFT polymerization of styrene [41]. Hence, the RAFT emulsion polymerizations discussed in the present work constitute straightforward routes to block copolymers, which are hardly obtainable utilizing other synthetic methods.

The in situ-formed micelles during the emulsion polymerizations were additionally investigated by DLS and cryoTEM, which has revealed well-defined particle sizes, as well as thermosensitive coronas indeed collapsing at the PTT of the thermoresponsive block, though in a rather gradual fashion. To the best of our knowledge, this is hence one of the first reports on surfactant-free RAFT emulsion polymerizations dealing with the synthesis of styrenic high-molecular-weight block copolymers containing thermoresponsive coronal blocks and investigating their temperature-dependent behavior in detail. Another option we could imagine for that purpose is photoinitiated PISA, since it allows for low polymerization temperatures maintaining the hydrophilicity of the thermoresponsive macroRAFT agent [16,26]. However, the polymerization rate of styrene under these conditions should be very low, rather limiting the feasibility of this option to faster propagating hydrophobic monomers.

We think that the presented methods are versatile and easily reproducible tools in both synthetic concerns and for creating ‘smart’ nano-objects with properties being tailored according to their envisaged application. In fact, different PS latexes generated by emulsion polymerization have already been tested in the biomedical area, e.g., for their protein adsorption [38], as microRNA delivery vectors [70], for the expansion and release of stem cells [32] or for their biocompatibility [43], and we could imagine applications in similar fields for our systems. We are nevertheless aware that more research is necessary to fulfill the requirements for materials being used in the medical or biological area. One issue is certainly the systems’ cytotoxicity and the removal of the DOX.

Another important aspect of this work is the absence of low-molecular-weight surfactants being difficult to separate after the polymer synthesis. Upscaling of the syntheses including the recycling of the solvents should hence be possible and is one of our next steps to deliver a sustainable large-scale procedure for creating high-molecular-weight smart polymers.

## Figures and Tables

**Table 1:** Experimental and analytical data of the PAPy-*b*-PS samples synthesized by RAFT emulsion polymerization and of the used PAPy macroRAFT agents. The key for the sample codes can be found in Figure 1. Weight fractions were determined by ^1^H NMR as shown in Figure S1c. DTPA, 2-(dodecylthiocarbonothioylthio)propionic acid.

Sample code	Used RAFT agent	Used styrene fraction ^a^ (wt %)	[Monomer]/[RAFT]	Polymerization time (min)	Monomer conversion (%)	${\bar{M}}_{n,\mathrm{th}}$ ^b^ (kDa)	${\bar{M}}_{n,\mathrm{app}}$ (kDa)	*Ð*
Y^9.6^	DTPA	–	156 ^c^	45	47	9.6	4.4 ^d^	1.36 ^d^
Y^12^	DTPA	–	157 ^c^	60	58	12	5.5 ^d^	1.33 ^d^
Y^18^	DTPA	–	317 ^c^	105	44	18	12 ^d^	1.29 ^d^
Y_20_S_80_^37^	Y^9.6^	82	416 ^e^	360	87	47	37 ^f^	1.30 ^f^
Y_18_S_82_^49^	Y^12^	82	514 ^e^	260	98	64	49 ^f^	1.35 ^f^
Y_32_S_68_^42 g^	Y^18^	69.5	398 ^e^	210	96	58	42 ^f^	1.29 ^f^
Y_21_S_79_^77^	Y^18^	82	795 ^e^	300	98	99	77 ^f^	1.38 ^f^
Y_19_S_81_^78^	Y^18^	85.5	1040 ^e^	480	84	109	78 ^f^	1.34 ^f^
Y_14_S_86_^106^	Y^18^	87.5	1214 ^e^	450	96	139	106 ^f^	1.41 ^f^
Y_12_S_88_^110^	Y^18^	90	1589 ^e^	480	87	162	110 ^f^	1.92 ^f^
Y_7_S_93_^192^	Y^18^	95	3178 ^e^	480	83	293	192 ^f^	2.62 ^f^

^a^ Compared to PAPy macroRAFT agent; ^b^ Calculated by ${\bar{M}}_{n,\mathrm{th}}=\frac{\left[\mathrm{monomer}\right]}{\left[\mathrm{RAFT}\right]}\times M_{\mathrm{monomer}}\times \mathrm{monomer}\;\mathrm{conversion}+M_{\mathrm{RAFT}}$. *M*: molecular weight. *M*_RAFT_ = ${\bar{M}}_{n,\mathrm{th},\mathrm{macroRAFT}}$ in the case of using a macroRAFT agent; ^c^ APy used as monomer; ^d^ Determined by *N,N*-dimethylacetamide (DMAc)-size-exclusion chromatography (SEC) with PMMA calibration; ^e^ Styrene used as monomer; ^f^ Determined by DMAc-SEC with PS calibration; ^g^ H_2_O/DOX (7/3, *v*/*v*) was used as the polymerization medium due to the higher PAPy concentration of 30 g·L^−1^.

**Table 2:** Experimental and analytical data of the PDMA-*b*-P(APi-*co*-APy)-*b*-PS block copolymers synthesized by RAFT emulsion polymerization and of the used macroRAFT agents. The key for the sample codes can be found in Figure 1. Weight fractions were determined by ^1^H NMR as shown in Figure S1d.

Sample code	Used RAFT agent	[Monomer]/[RAFT]	Polymerization time (min)	Monomer conversion (%)	${\bar{M}}_{n,\mathrm{th}}$ ^a^ (kDa)	${\bar{M}}_{n,\mathrm{app}}$ (kDa)	*Ð*
D^2.6^	DTPA	30 ^b^	90	77	2.6	1.7 ^c^	1.24 ^c^
D_9_(I_100_)_91_^28^	D^2.6^	424 ^d^	145	43	28	18 ^c^	1.25 ^c^
D_12_(I_70_Y_30_)_88_^22^	D^2.6^	435 ^d^	77	33	22	14 ^c^	1.27 ^c^
D_10_(I_47_Y_53_)_90_^26^	D^2.6^	442 ^d^	72	40	26	17 ^c^	1.28 ^c^
D_9_(I_25_Y_75_)_91_^28^	D^2.6^	470 ^d^	67	42	28	18 ^c^	1.27 ^c^
D_11_(Y_100_)_89_^24^	D^2.6^	467 ^d^	67	37	24	14 ^c^	1.45 ^c^
D_2_(I_100_)_20_S_78_^110^	D_9_(I_100_)_91_^28^	1231 ^e^	180	97	152	110 ^f^	1.36 ^f^
D_2_(I_70_Y_30_)_19_S_79_^103^	D_12_(I_70_Y_30_)_88_^22^	971 ^e^	195	98	121	103 ^f^	1.43 ^f^
D_2_(I_47_Y_53_)_18_S_80_^116^	D_10_(I_47_Y_53_)_90_^26^	1150 ^e^	180	97	142	116 ^f^	1.64 ^f^
D_2_(I_25_Y_75_)_20_S_78_^88^	D_9_(I_25_Y_75_)_91_^28^	1214 ^e^	240	78	127	88 ^f^	1.57 ^f^
D_2_(Y_100_)_19_S_79_^93^	D_11_(Y_100_)_89_^24^	1066 ^e^	360	98	133	93 ^f^	1.53 ^f^

^a^ Calculated by ${\bar{M}}_{n,\mathrm{th}}=\frac{\left[\mathrm{monomer}\right]}{\left[\mathrm{RAFT}\right]}\times M_{\mathrm{monomer}}\times \mathrm{monomer}\;\mathrm{conversion}+M_{\mathrm{RAFT}}$. *M*: molecular weight. *M*_RAFT_ = ${\bar{M}}_{n,\mathrm{th},\mathrm{macroRAFT}}$ in case of using a macroRAFT agent; ^b^ DMA used as monomer; ^c^ Determined by DMAc-SEC with PMMA calibration; ^d^ APy and APi used as comonomers in different ratios (see Table S1); ^e^ Styrene used as monomer; ^f^ Determined by DMAc-SEC with PS calibration.

## Supplementary Materials

The following are available online at www.mdpi.com/2073-4360/9/12/668/s1: Figure S1: ^1^H NMR spectra; Figure S2: SEC data for the RAFT emulsion polymerization of styrene using Y^9.6^ as the PAPy macroRAFT agent/macro-stabilizer; Figure S3: Picture of the coagulum appearing in the RAFT emulsion polymerizations; Table S1: Initial molar ratios of APi and APy used to generate the diblock copolymer macroRAFT agents/macro-stabilizers via the nanoreactor approach; Figure S4: SEC traces of the other four PDMA-*b*-P(APi-*co*-APy)-*b*-PS triblock copolymers being obtained by the combination of cosolvent and nanoreactor approach; Figure S5: Exemplary and representative heating–cooling cycles from temperature-dependent DLS measurements of three aqueous micellar solutions obtained in the different emulsion polymerizations; Figures S6–S11: Further cryoTEM images of the latexes.
