# Supplementary material for: Surfactant-Free RAFT Emulsion Polymerization of Styrene Using Thermoresponsive macroRAFT Agents: Towards Smart Well-Defined Block Copolymers with High Molecular Weights
Source: Polymers (Basel). 2017 Dec 3;9(12):668. doi: 10.3390/polym9120668 (PMC6418535; doi:10.3390/polym9120668)
Supplement: Supplementary file 1 [file polymers-09-00668-s001.pdf]

Supplementary Information

Surfactant-Free RAFT Emulsion Polymerization of Styrene Using Thermoresponsive macroRAFT Agents: Towards Smart Well-Defined Block Copolymers with High Molecular Weights

Steffen Eggers [1], Volker Abetz [1,2,*]

[1] University of Hamburg, Department of Physical Chemistry, Grindelallee 117, 20146 Hamburg, Germany; steffen.eggers@chemie.uni-hamburg.de

[2] Helmholtz-Zentrum Geesthacht, Institute of Polymer Research, Max-Planck-Straße 1, 21502 Geesthacht, Germany; volker.abetz@hzg.de

[*] Correspondence: volker.abetz@hzg.de; Tel.: +49-42838-3460

^{1}H NMR Spectra

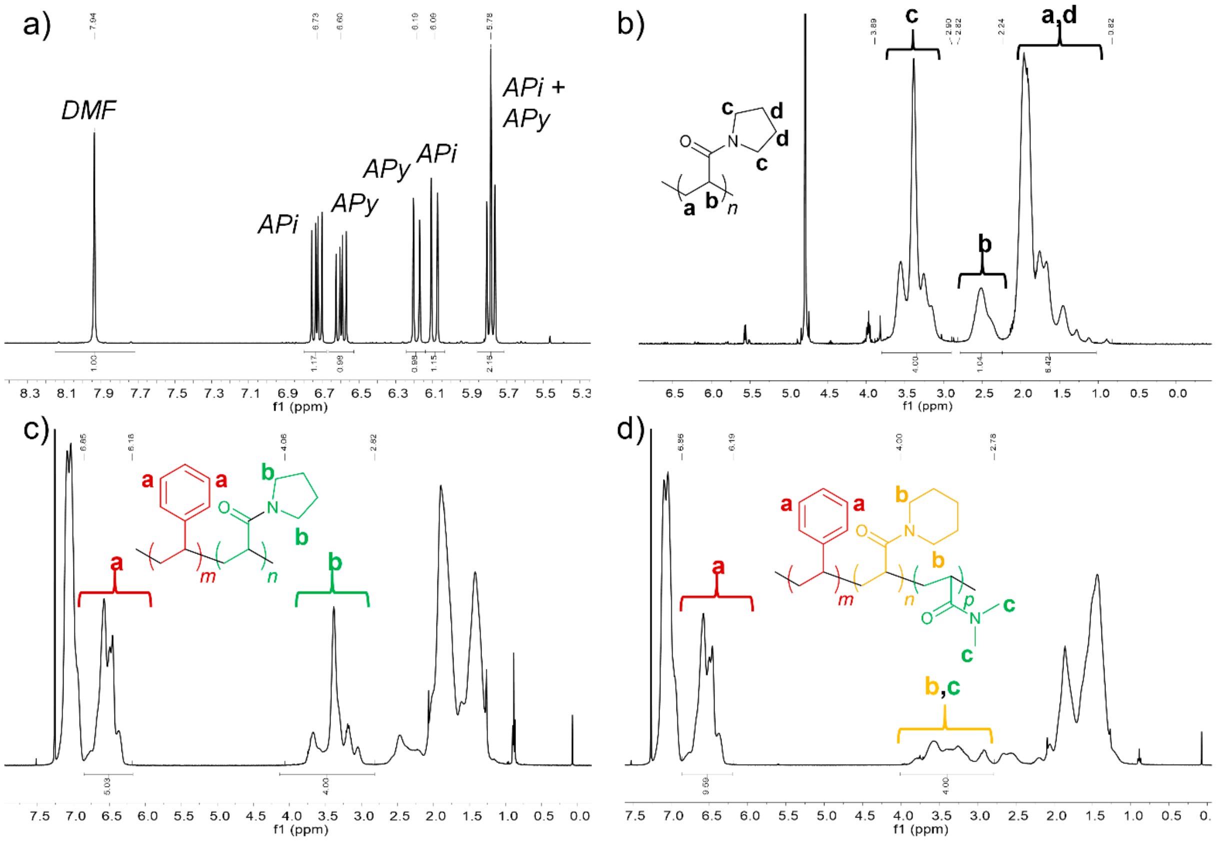

Figure S1. ^{1}H NMR spectra. (**a**) Determination of the monomer conversion in the copolymerizations of APi and APy from the crude polymerization mixture by calculating the integral ratio of the DMF formyl signal (as reference) and the combined monomer signal at 5.78 ppm before and after polymerization. The spectrum was recorded in D_2O. (**b**) ^{1}H NMR spectrum of PAPy in D_2O. (**c**) ^{1}H NMR spectrum of PAPy-*b*-PS in $CDCl_3$ to determine the block weight fractions. (**d**) ^{1}H NMR spectrum of PDMA-*b*-P(APi-*co*-APy)-*b*-PS in $CDCl_3$ to determine the block weight fractions.

RAFT Emulsion Polymerization Using the Shorter PAPy macroRAFT Agent/Macro-Stabilizer $Y^{9.6}$

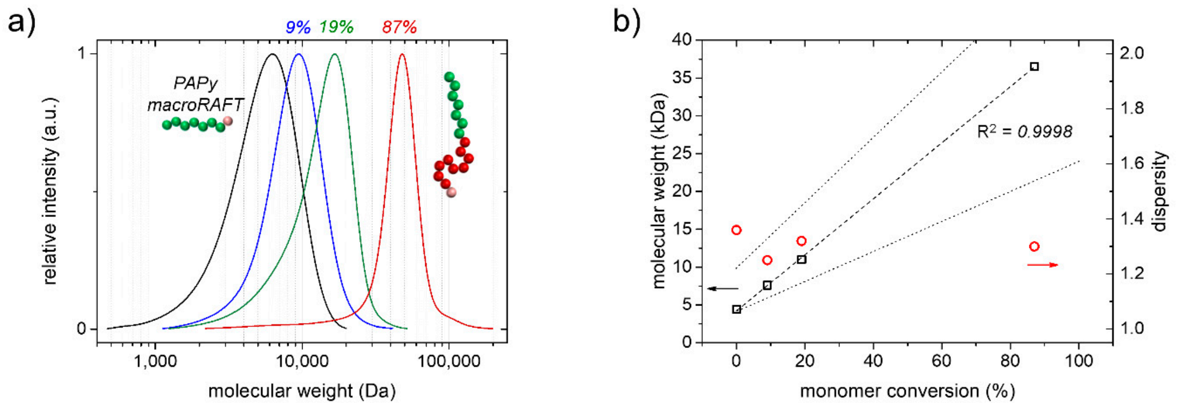

Figure S2. SEC data for the RAFT emulsion polymerization of styrene using $Y^{9.6}$ as the PAPy macroRAFT agent/macro-stabilizer. (**a**) Evolution of the SEC traces with styrene conversion (values indicated). (**b**) Apparent number-average molecular weights ($\bar{M}_{n,app}$, left ordinate, black squares) and dispersities (right ordinate, red circles) in dependence on the styrene conversion. The molecular weights are linearly fitted, the regression coefficient R^2 of the fit is given. Furthermore indicated are the theoretical values calculated with $\bar{M}_{n,th,macroRAFT}$ (upper dotted line) and $\bar{M}_{n,app,macroRAFT}$ (lower dotted line), respectively.

Amount of Formed Coagulum During the Emulsion Polymerizations

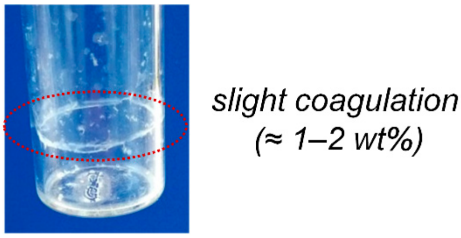

Figure S3. Picture of the coagulum (marked by a red circle) appearing in the RAFT emulsion polymerizations. The total solids amount in the formulation was in this case ca. 110 mg, the polymer yield was ca. 100 mg, the amount of coagulum was ca. 1–2 mg.

Used APi/APy Ratios to Synthesize the PDMA-*b*-P(APi-*co*-APy) macroRAFT Agents/Macro-Stabilizers

Table S1. Initial molar ratios of APi and APy used to generate the diblock copolymer macroRAFT agents/macro stabilizers *via* the nanoreactor approach. The comonomer ratios in the polymer can be deduced from the sample codes (see Figure 1). The reason for the slightly higher APy fraction in the polymer than in the feed is its slightly higher reactivity ratio (*r*) compared to APi (r_{APy} = 1.24, r_{APi} = 0.84) [1]. This is discussed in more detail elsewhere [1].

Sample code	Initial molar APi/APy ratio
$D_9(I_{100})_{91}^{28}$	100/0
$D_{12}(I_{70}Y_{30})_{88}^{22}$	74/26
$D_{10}(I_{47}Y_{53})_{90}^{26}$	52/48
$D_9(I_{25}Y_{75})_{91}^{28}$	28/72
$D_{11}(Y_{100})_{89}^{24}$	0/100

Further SEC Traces of the Synthesized Triblock Copolymers

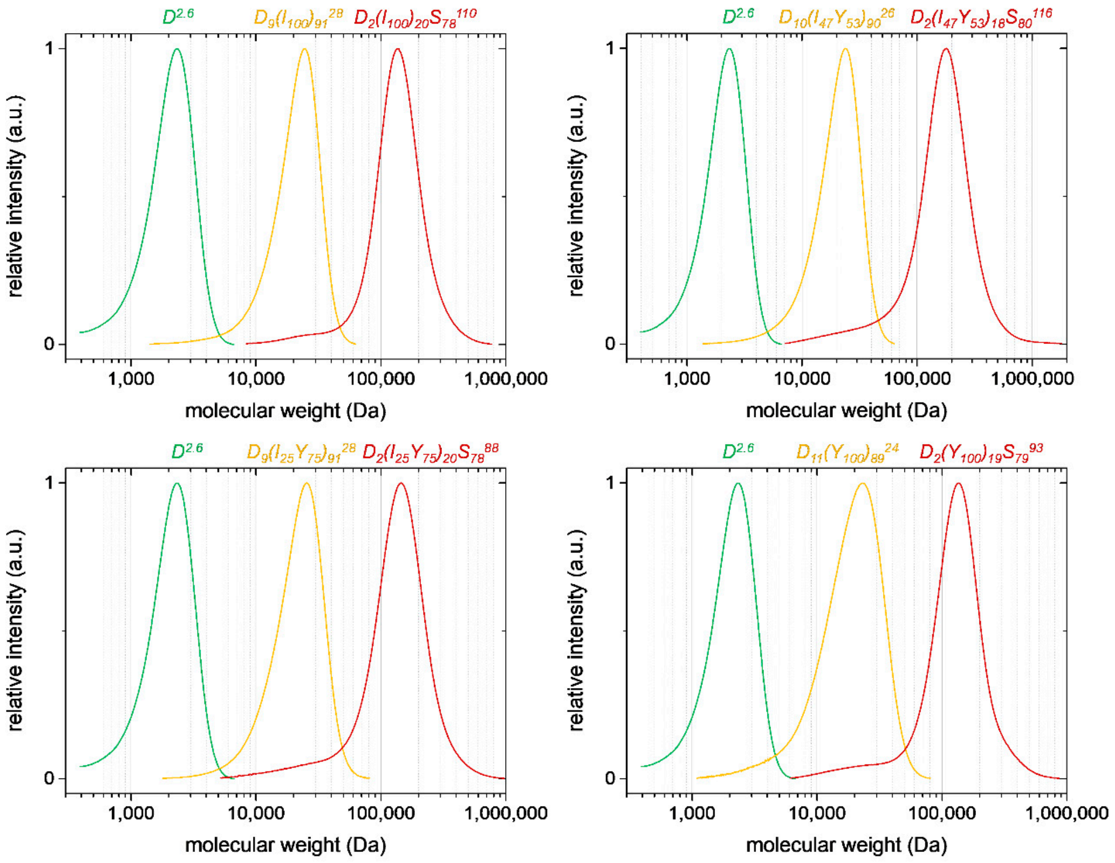

Figure S4. SEC traces of the other four PDMA-*b*-P(APi-*co*-APy)-*b*-PS triblock copolymers being obtained by the combination of cosolvent and nanoreactor approach. A key for the sample codes is given in Figure 1, more analytical data can be found in Table 2.

Exemplary Data for the Reversibility of the Temperature-Induced Corona Collapse

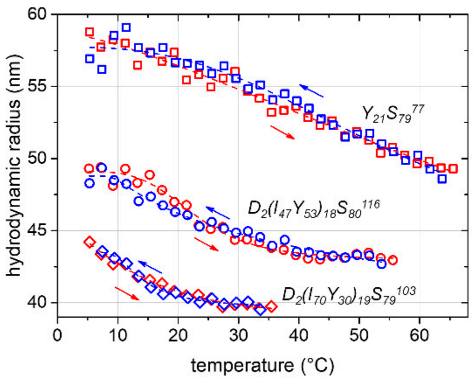

Figure S5. Exemplary and representative heating-cooling cycles from temperature-dependent DLS measurements of three aqueous micellar solutions obtained in the different emulsion polymerizations. The heating is depicted in red, the cooling in blue. The hydrodynamic radius was evaluated by cumulant fitting.

Further CryoTEM Images of the Latexes

$Y_{21}S_{79}^{77}$

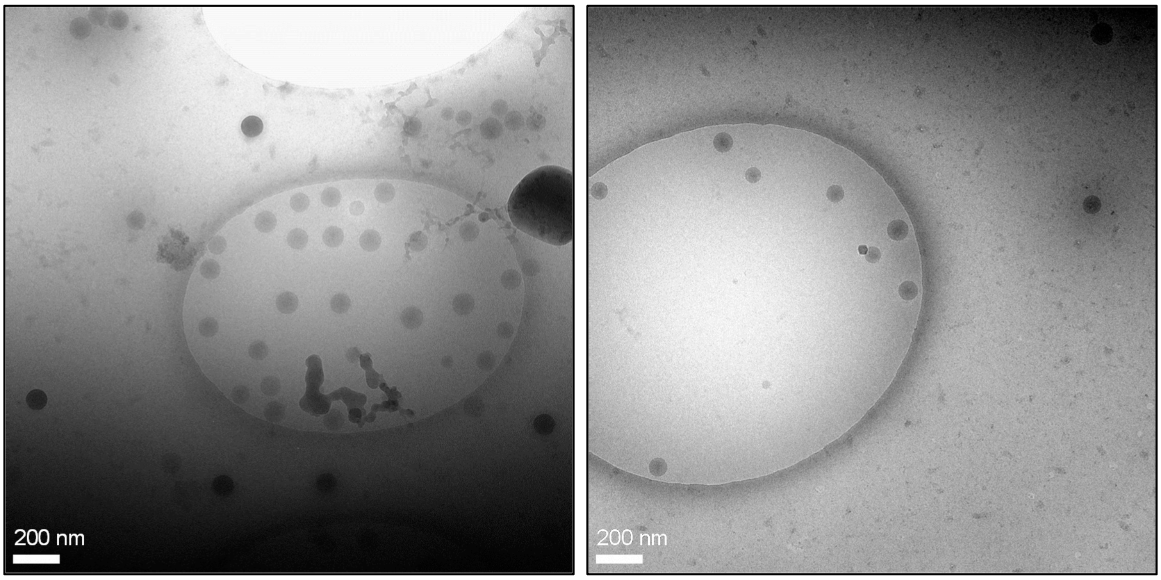

Figure S6. CryoTEM images of the micelles of sample $Y_{21}S_{79}^{77}$ in water.

$Y_{14}S_{86}^{106}$

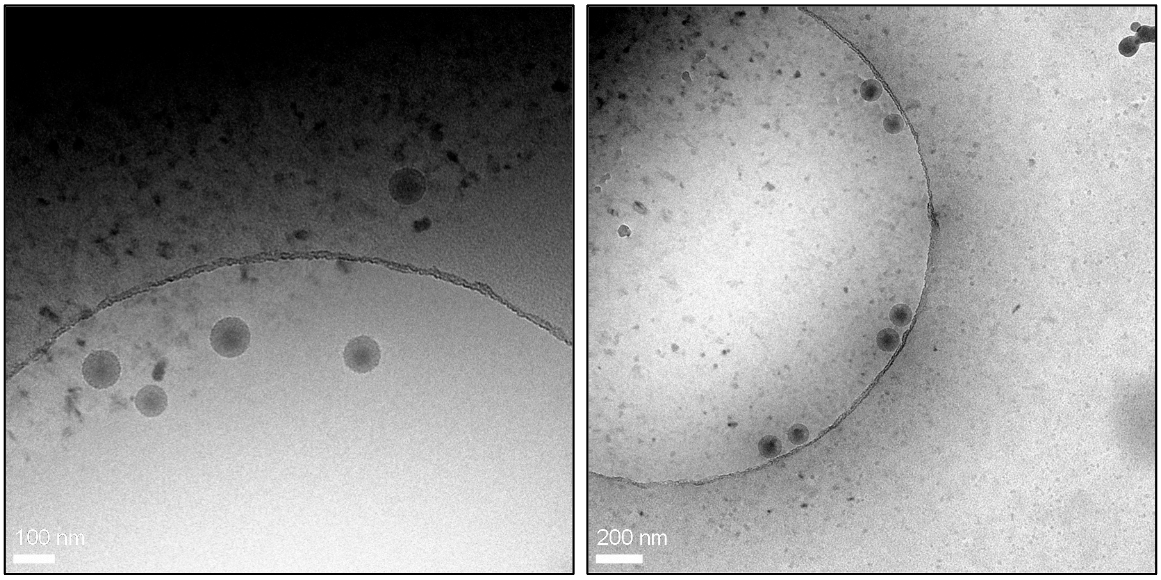

Figure S7. CryoTEM images of the micelles of sample $Y_{14}S_{86}^{106}$ in water.

$Y_{12}S_{88}^{110}$

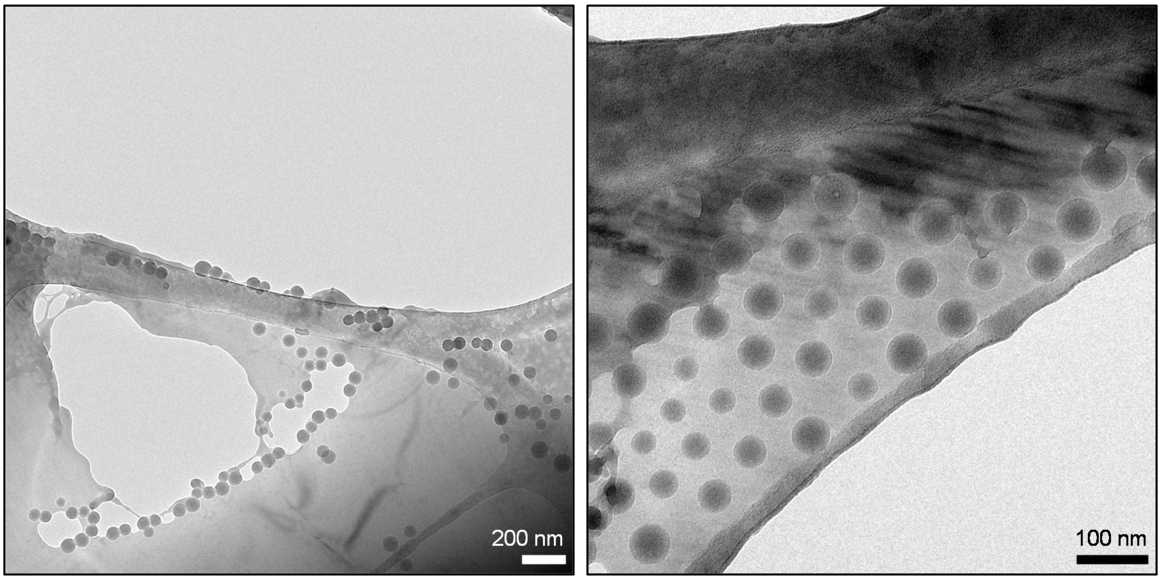

Figure S8. CryoTEM images of the micelles of sample $Y_{12}S_{88}^{110}$ in water.

$Y_{7}S_{93}^{192}$

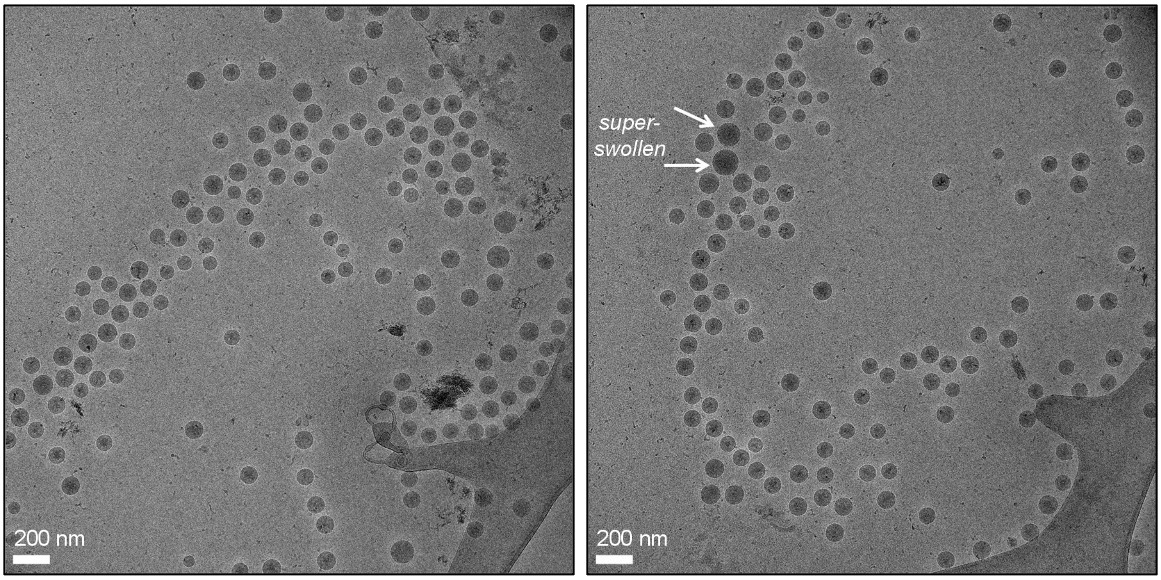

Figure S9. CryoTEM images of the micelles of sample $Y_{7}S_{93}^{192}$ in water. In the right image, two 'unsplit' micelles are marked, probably being the reason for the molecular weight broadening of this sample.

$D_2(I_{25}Y_{75})_{20}S_{78}^{88}$

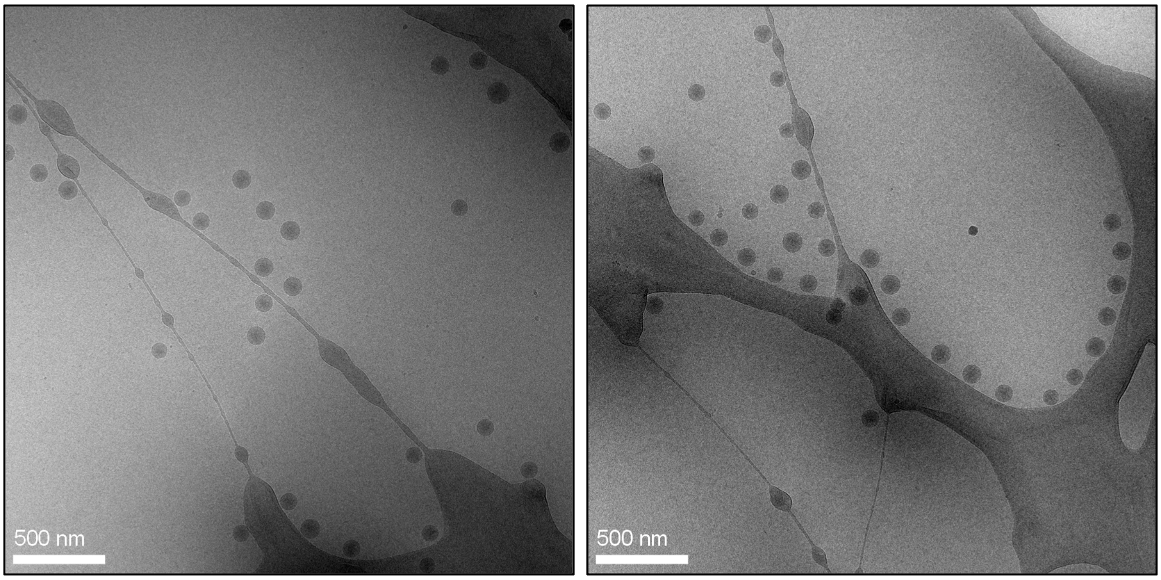

Figure S10. CryoTEM images of the micelles of sample $D_2(I_{25}Y_{75})_{20}S_{78}^{88}$ in water.

$D_2(I_{70}Y_{30})_{19}S_{79}^{103}$

*Vitrobot at 4 °C (below the PTT of the P(APi-*co*-APy) block)*

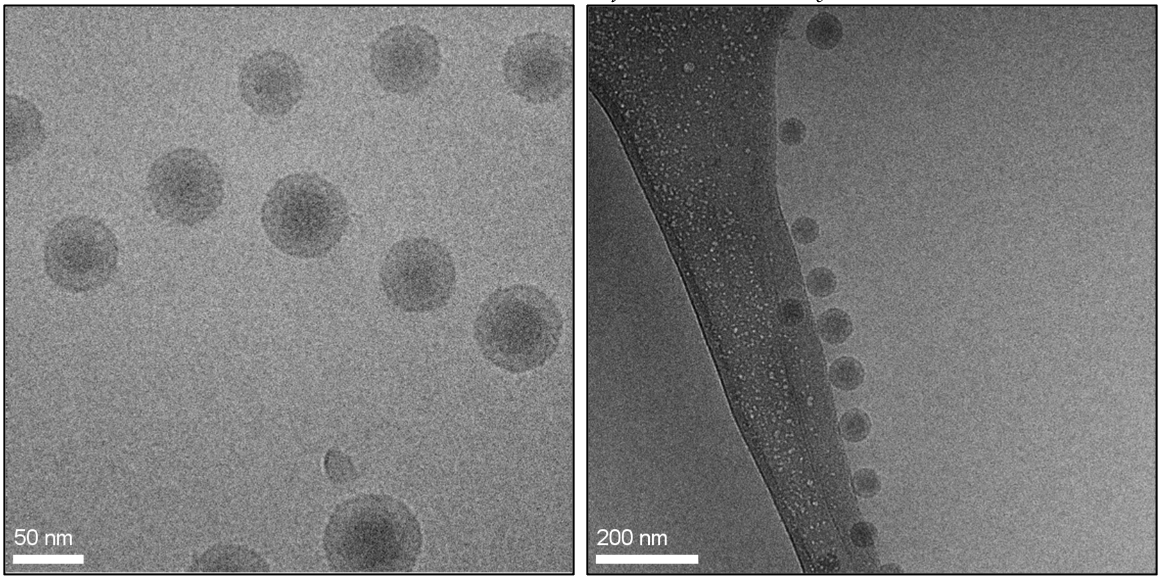

*Vitrobot at 30 °C (above the PTT of the P(APi-*co*-APy) block)*

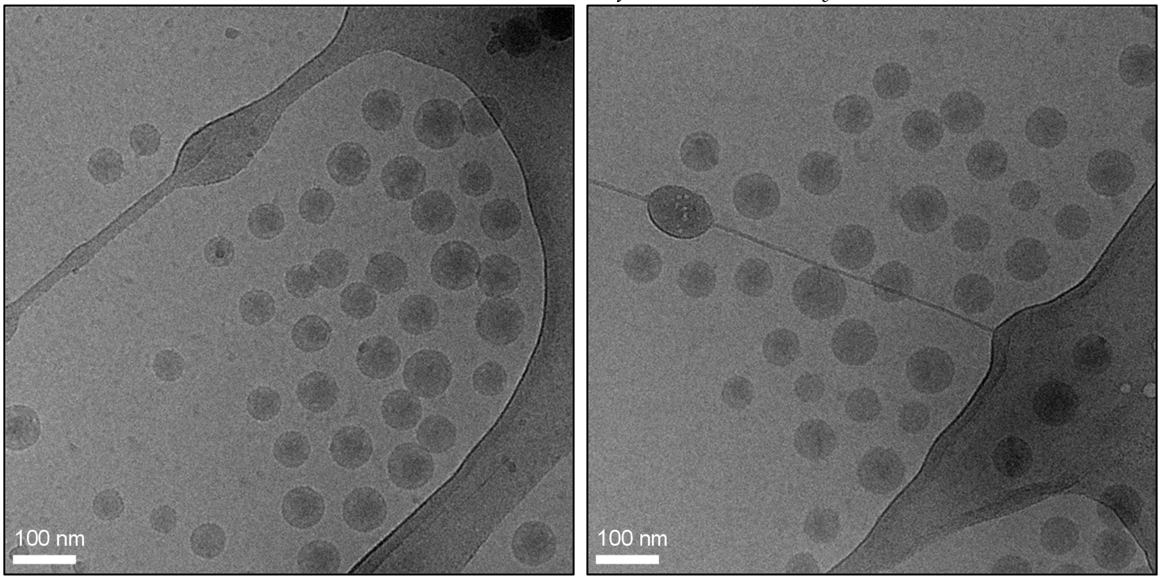

Figure S11. CryoTEM images of the micelles of sample $D_2(I_{70}Y_{30})_{19}S_{79}^{103}$ in water at different temperatures.

References

1. Eggers, S.; Eckert, T.; Abetz, V. Double thermoresponsive block–random copolymers with adjustable phase transition temperatures: From block-like to gradient-like behavior. *J. Polym. Sci., Part A: Polym. Chem.* **2017**, doi:10.1002/pola.28906.
